# Allergen Delivery Inhibitors: A Rationale for Targeting Sentinel Innate Immune Signaling of Group 1 House Dust Mite Allergens through Structure-Based Protease Inhibitor Design

**DOI:** 10.1124/mol.118.112730

**Published:** 2018-09

**Authors:** Jihui Zhang, Jie Chen, Gary K. Newton, Trevor R. Perrior, Clive Robinson

**Affiliations:** Institute for Infection and Immunity, St George’s, University of London, London, United Kingdom (J.Z., J.C., C.R.); State Key Laboratory of Microbial Resources, Institute of Microbiology, Chinese Academy of Sciences, Beijing, People’s Republic of China (J.Z.); and Domainex Ltd., Chesterford Research Park, Saffron Walden, United Kingdom (G.K.N., T.R.P.)

## Abstract

Diverse evidence from epidemiologic surveys and investigations into the molecular basis of allergenicity have revealed that a small cadre of “initiator” allergens promote the development of allergic diseases, such as asthma, allergic rhinitis, and atopic dermatitis. Pre-eminent among these initiators are the group 1 allergens from house dust mites (HDM). In mites, group 1 allergens function as cysteine peptidase digestive enzymes to which humans are exposed by inhalation of HDM fecal pellets. Their protease nature confers the ability to activate high gain signaling mechanisms which promote innate immune responses, leading to the persistence of allergic sensitization. An important feature of this process is that the initiator drives responses both to itself and to unrelated allergens lacking these properties through a process of collateral priming. The clinical significance of group 1 HDM allergens in disease, their serodominance as allergens, and their IgE-independent bioactivities in innate immunity make these allergens interesting therapeutic targets in the design of new small-molecule interventions in allergic disease. The attraction of this new approach is that it offers a powerful, root-cause-level intervention from which beneficial effects can be anticipated by interference in a wide range of effector pathways associated with these complex diseases. This review addresses the general background to HDM allergens and the validation of group 1 as putative targets. We then discuss structure-based drug design of the first-in-class representatives of allergen delivery inhibitors aimed at neutralizing the proteolytic effects of HDM group 1 allergens, which are essential to the development and maintenance of allergic diseases.

## Introduction

Over the past 40 years, pharmacology, immunology, and cell biology have contributed enormously to understanding the mechanisms of diseases with allergic etiologies, viz. atopic dermatitis, allergic rhinitis, and much of asthma. The pharmacologic contribution to this research effort has created a wealth of new molecular and biologic entities that have enjoyed success as experimental tools. However, for new entities acting upon unprecedented targets, this drug discovery enterprise has largely failed when assessed against the metric of clinical success. These endeavors have revealed the inherent weakness in treating the effects rather than causes of disease and have demonstrated convincingly the difficulties faced when making focused interventions against strategic effector mechanisms that are backed up by multiple redundancies. Indeed, the supremacy of inhaled corticosteroids as a standard of care for asthma highlights the benefits of a broad spectrum of action, but despite this, discrete targets in downstream effector pathways continue to attract attention. The more recent focus on interventions based on new biologic entities, such as monoclonal antibodies directed against interleukin 5 (IL-5), the shared IL-4/IL-13 receptor *α*-subunit, or thymic stromal lymphopoietin (TSLP), has demonstrated that success with new approaches is certainly feasible when patients are rigorously stratified for treatment. Nevertheless, the overarching philosophy in these newly approved or emerging biologic approaches remains directed at downstream effectors, and inevitably, the reminders of the high risk of failure associated with this level of intervention are ever present because not all anticytokine development programs have enjoyed positive outcomes in asthma. This raises strategic questions for the design of new approaches to these diseases, and among the most challenging topics are the identification of tractable new targets and the future that remains for small-molecule design in an era when biologics are in the limelight.

Encouragingly, alternative philosophies have delivered success, as demonstrated by omalizumab, the monoclonal antibody therapy which depletes circulating IgE to eventually reduce the amount of antibody bound to high-affinity IgE receptors on mast cells. The development of omalizumab represented a significant change from a focus on symptom amelioration to an upstream intervention potentially capable of disease modification. However, among other significant issues, biologic therapies such as omalizumab pose significant reimbursement challenges which are problematic for patient access to treatment in chronic diseases affecting large populations.

From a theoretical perspective, an ideal intervention would target a root cause of allergic disease, namely, allergens themselves. This is, of course, partly the basis of the long-standing practice of allergen-specific immunotherapy. For small-molecule interventions, this root-cause approach has been generally viewed as an unattainable goal: there are many allergens in the environment, many of which remain poorly characterized, and exactly why they are allergenic—that is, break immune tolerance and promote IgE-directed immune responses—has, until relatively recently, been a surprisingly unfashionable topic in allergy. However, progress in the characterization of allergens has shed new light on the molecular basis of allergenicity, and this has coincided with a renaissance of interest in innate immunity. This creates the possibility of making interventions at critical checkpoints in these pathways to prevent the development of allergic disease. In particular, it is now possible to design an approach to target a major asthma trigger at root-cause level, with the advantage that this circumvents concerns about the chronic consequences of blocking checkpoints which are of fundamental importance to immunity. Unlike allergen-specific immunotherapy, an important advantage of this new line of attack is the prevention of innate immune responses which are the engine of allergy development. The purpose of this review is to examine the biologic mechanisms by which a major house dust mite (HDM) allergen triggers allergic disease, and describe the design of an intervention to specifically neutralize this activity. The choice and nature of the target are unprecedented and may be unfamiliar to those outside the immediate field; therefore, as an aid to interested newcomers, we also describe the historical background to HDM allergens and allergic disease, their clinical significance, and a brief overview of the complete spectrum of denominated HDM allergens.

## HDM Allergens and Disease

House dust is a complex mixture of biologically diverse components from various sources with properties that render it inhalable by humans. House dust as a cause of allergic disease was first recognized in 1921 by the American physician Richard Kern, who observed that many patients with rhinitis or asthma had positive skin reactions to extracts of dust from their own homes. This idea was elaborated further in the following two decades by Willem Storm van Leeuwen, Professor of Pharmacology and Director of the Pharmacotherapeutic Institute at the University of Leiden, who proposed that the cause of asthma could be the presence of mites in house dust. However, conclusive proof remained elusive until 1967, when Spieksma and Voorhorst and their colleagues (Voorhorst et al., 1967) established that *Dermatophagoides pteronyssinus* was an important source of indoor allergens—a linkage which we now regard as intuitive, but which at the time was received with fierce skepticism from many allergists. It is now recognized that HDMs such as *D. pteronyssinus* or *Dermatophagoides farinae* form the single most important indoor allergen source associated with asthma in temperate climates and lead to the development of high-titer allergen-specific IgE. The growth in sensitization of human populations is, in part, promoted by indoor, sedentary, affluent lifestyles and the creation of warm, humid, draft-free habitation and working conditions which provide optimal growth conditions for HDMs. Consequently, many people are extensively exposed to HDMs with the result that allergic diseases triggered by indoor allergens are major healthcare problems with a significant socioeconomic impact.

Substantial evidence associates allergic conditions such as asthma, chronic rhinitis, atopic dermatitis, and, less frequently, conjunctivitis with exposure to HDM or other indoor allergens (Smith et al., 1969; Platts-Mills et al., 1987, 1997; Sporik et al., 1990; Gelber et al., 1993; Peat et al., 1996; Squillace et al., 1997; Pichavant et al., 2005; Platts-Mills, 2009). Data from longitudinal investigations suggest that the development of sensitization to HDM occurs before polysensitization (Silvestri et al., 1999; Purello-D’Ambrosio et al., 2001), that is, the development of allergy to other triggers, a phenomenon which occurs in ∼50%–80% of people (Calderón et al., 2012). At first, this ordering of events in polysensitization seems puzzling until it is recognized that the bioactivity of certain allergens makes them unusually potent stimulants and providers of collateral immune priming on which other allergens depend. In this regard, certain HDM proteins function broadly as bioinitiators as well as simply being allergenic.

HDMs are small arthropods, ∼250 *µ*m in length, related to spiders and scorpions. Many mite species are found in house dust, but the pyroglyphid family dominates in most areas of the world (e.g., *D. pteronyssinus, D. farinae*, and *Euroglyphus maynei*). In the tropics, allergy to *Blomia tropicalis* may also be prevalent. Dust mites live on a diet of exfoliated human skin flakes and other biodebris. Existing on this diet means that HDMs have digestive enzymes which can process tough structural proteins. Food waste is excreted, together with the digestive enzymes, in pellets which are of a respirable aerodynamic diameter (10–30 *µ*m). As described later, these excreted digestive enzymes are significant because they are allergenic with a bioactivity profile which provides them with the ability to promote sensitization to themselves and unrelated inhalant allergens, regardless of source. Sensitization to HDM allergens occurs through inhalation of their fecal pellets, which impact on the airway epithelium where airway surface liquid triggers the release of the pellet contents, resulting in a high concentration of allergen at the site of deposition. Sensitization by inhalation may also be the case in people who develop atopic dermatitis rather than allergic conditions of the airways, although in established dermatitis, any ensuing allergic responses are more likely to result from skin contact.

### 

#### Allergen Nomenclature.

Allergens are classified according to a system devised in 1984 by the Allergen Nomenclature Sub-Committee, which was established jointly by the World Health Organization and the International Union of Immunologic Societies. The systematic naming of allergens takes the accepted binomial nomenclature of the source and uses the first three letters of the genus combined with the first one or two letters of the species name followed by an Arabic numeral reflecting the order in which the allergen was isolated or its clinical importance (or, in reality, a hybrid of both). This numbering allows the denomination of allergens by group. In the case of *Dermatophagoides pteronyssinus*, the systematic naming takes the form Der p X, where X is the group number, and this is replicated in similar allergens from related species. Thus, HDM cysteine protease allergens from *D. pteronyssinus*, *D. farinae*, *Euroglyphus maynei*, and *Blomia tropicalis* are known individually as Der p 1, Der f 1, Eur m 1, and Blo t 1, respectively; collectively as the group 1 HDM allergens; or as the HDM cysteine proteases. Denomination is further resolved to accommodate the allelic variation in proteins, which gives rise to isoallergens. Those allergens from the same source species with >67% sequence identity are denoted using suffixes which may be up to four digits in length according to the complexity of the isoallergenic variations (e.g., Der p 1.0101, Der p 1.0102, etc.). As an additional refinement, it is sometimes necessary to denote whether allergens are in their native (n) forms or are products of recombinant protein engineering (r). Recombinant allergens differ most notably in protein glycosylation, which is usually due to the need to modify potential glycosylation sites, e.g., to prevent hyperglycosylation of proteins expressed in yeast, an expression system which is often favored because it generally provides correct protein folding and thus mimics conformational epitopes and other biologic characteristics seen in native allergens. Whereas some glycosylation changes may have neutral or little impact on bioactivities such as the protease behavior of Der p 1 (Zhang et al., 2009), they are more relevant to the processing of allergens by antigen-presenting cells. Thus, the prefixes n and r, respectively, may be appended (e.g., nDer p 1, rDer p 1).

More than 30 groups of HDM allergens have now been denominated, making HDMs probably the single largest source of allergens to which humans are exposed. These allergens do not, however, have equal clinical importance because a clear serodominance is observable for those belonging to groups 1 and 2. A summary of HDM allergens and their properties is provided in Table 1. Molecular biology, genomics, and bioinformatics have played key roles in the elucidation of these groups, particularly in predicting their functional properties in the host—information which provides important insights into why those molecules behave as allergens in humans. Further information about the characteristics of these allergen groups can be obtained by using the GenBank and UniProt accession codes listed in Table 1.

**TABLE 1:** Group denominations of HDM allergens, with exemplars, and their bioactivity profiles based on bioinformatics predictions and/or empirical observations The list omits storage mite species exemplars of the subclass Acari, although in many cases their allergens show the same grouping and characteristics as the HDM allergens. Exemplar GenBank and UniProt accession codes of the listed allergens are provided for supporting literature references and bioinformatics substantiation (e.g., by hyperlink to simple modular architecture research tool “SMART”), but codes for isoallergenic variants are omitted for brevity.

HDM Allergen Group Exemplars	Molecular Mass	Known or Predicted Bioactivity	GenBank/EMBL	UniProt
	*kDa*			
Group 1, e.g., Der p 1	25 (39 Blo t 1)	Cysteine protease	U11695	P08176
Der f 1			AB03496	Q58A71
Eur m 1			AF047610	P25780
Der m 1			—	P16312
Blo t 1			AF277840	Q95PJ4
Group 2, e.g., Der p 2	14	NPC2 family; MD-2–related protein, lipid binding, binds LPS	AF276239	P49278
Der f 2			D10447	Q00855
Eur m 2			AF047613	Q9TZZ2
Blo t 2			AY288141	Q1M2P1
Group 3, e.g., Der p 3	25	Trypsin	U11719	P39675
Der f3			D63858	P49275
Eur m3			AF047615	O97370
Blo t 3			AY291323	A1KXI1
Group 4, e.g., Der p 4	60	Amylase	AF144060	Q9Y197
Der f 4			KM016832	A0A089FLV3
Eur m 4			AF144061	Q9Y196
Blo t 4			AY291324	A1KXI2
Group 5, e.g., Der p 5	14	Function unknown; ligand-binding protein?	S76337	P14004
Blo t 5			U59102	O96870
Group 6, e.g., Der p 6	25	Chymotrypsin	—	P49277
Der f 6			AF125187	P49276
Blo t 6			AY291325	A1KXI3
Group 7, e.g., Der p 7	26–31	Bactericidal permeability-increasing like protein of unknown function; belongs to the juvenile hormone binding family of proteins found in insects; may have lipid-binding properties	U37044	P49273
Der f 7			S80655	Q26456
Blo t 7			MF740745	—
Group 8, e.g., Der p 8	27	Glutathione *S*-transferase	S75286	P46419
Der f 8			KC305499	L7V2G7
Blo t 8			GQ398117	C8CGT7
Group 9, e.g., Der p 9	29	Collagenase-like serine protease	AY211952	Q7Z163
Group 10, e.g., Der p 10	36	Tropomyosin	Y14906	O18416
Der f 10			D17682	Q23939
Blo t 10			EU106615	A7XZI4
Group 11, e.g., Der p 11	103	Paramyosin	AY189697	Q6Y2F9
Der f 11			AF352244	Q967Z0
Blo t 11			AF525465	Q8MUF6
Group 12, e.g., Blo t 12	14	Possible chitinase; shows homology with Der f 15 due to chitin-binding domain	U27479	Q17282
Group 13, e.g., Der p 13	15	Fatty acid–binding protein	HM560018	E0A8N8
Der f 13			AY283293	Q1M2P5
Blo t 13			U58106	Q17284
Group 14, e.g., Der p 14	177	Vitellogenin or lipophorin	AF373221	Q8N0N0
Der f 14			D17686	Q94507
Eur m14			AF149827	Q9U785
Group 15, e.g., Der p 15	98, 109*^a^*	GH18 superfamily chitinase; shows homology with mite group 18.	DQ078741	Q4JK69
Der f 15			AF178772	Q9U6R7
Group 16, e.g., Der f 16	53	Gelsolin/villin	AF465625	Q8MVU3
Group 17, e.g., Der f 17	30	Calcium-binding protein	—	—
Group 18, e.g., Der p 18	60	GH18 superfamily chitinase; homologous with group 15	DQ078739	Q4JK71
Der f 18			AY093656	Q86R84
Group 19, e.g., Blo t 19	7	Antimicrobial peptide homology	KF771884	W5RZ24
Group 20, e.g., Der p 20	40	Arginine kinase	EU684970	B2ZSY4
Der f 20			KM009994.1	—
Group 21, e.g., Der p 21	14	Function unknown; shows homology with group 5 allergens	DQ354124	Q2L7C5
Der f 21			KF732965.1	B2GM84
Blo t 21			DQ788679	A7IZF1
Group 22, e.g., Der f 22	17	Shows homology with group 2 mite allergen; belongs to MD-2–related lipid recognition domain family; implicated in lipid binding	DQ643992	A5X5X4
Group 23, e.g., Der p 23	14–19	Unknown function; shows homology with peritrophin-A domain and contains a chitin-binding domain	EU414751.1	L7N6F8
Der f 23			KU166910	—
Group 24, e.g., Der p 24	13	Ubiquinol-cytochrome c reductase binding protein-like protein	KP893174	A0A0K2GUJ4
Der f 24			KC669700	M9RZ95
Group 25, e.g., Der f 25	34	Triosephosphate isomerase	KC305500.1	L7UZA7
Group 26, e.g., Der f 26	18	Myosin alkali light chain	KM009996*^b^*	A0A088SAG5*^b^*
Group 27, e.g., Der f 27	48	Serpin–trypsin inhibitor	AIO08851	—
Group 28, e.g., Der f 28	70	Heat shock protein	KC305502	L7V065
Group 29, e.g., Der f 29	16	Peptidyl-prolyl *cis-trans* isomerase (cyclophilin)	AY283280.1	A1KXG2
Group 30, e.g., Der f 30	16	Ferritin	KC305503	L7UZ91
Group 31, e.g., Der f 31	15	Cofilin, actin binding protein	KM010014	A0A088SAY1
Group 32, e.g., Der f 32	35	Secreted inorganic pyrophosphatase	KM009993	A0A088SCP3
Group 33, e.g., Der f 33	52	*α*-tubulin	KM010005	A0A088SV41
Group 34, e.g., Der f 34	16	Enamine/imine deaminase	LC120618	A0A1J1DL12
Group 35, e.g., Der f 35	14	MD-2–related protein homologous with group 2	LC175222	A0A1W7HBY9
Group 36, e.g., Der p 36	42	Function unknown; contains a C-terminal C2 domain (pfam00168) associated with signal transduction	KY465507	A0A291KZD3
Der f 36			KY465506	A0A291KZC2
Group 37, e.g., Der p 37	30	Peritrophic-like protein domain	MG520330*^c^*	—

EBML, European Molecular Biology Laboratory.

^a^ Glycosylated forms of 63-kDa protein predicted by DNA sequence.

^b^ Note that GenBank/European Bioinformatics Institute and UniProt incorrectly designate this as a group 30 allergen. Definitively, it is a group 26 allergen as designated by the Allergen Nomenclature Sub-Committee of the World Health Organization and International Union of Immunologic Societies. Please refer to http://www.allergen.org/viewallergen.php?aid=815 for further information.

^c^ Accession not yet published.

## Group 1 HDM Allergens as a Drug Design Target

As clan CA, C1 family cysteine proteases, group 1 HDM allergens have a basic structure, comprising a substrate-binding groove separating two globular domains, which is archetypally defined by the extensively studied representatives, papain, actinidin, and cathepsin K. Proteolytic latency of the HDM allergens is maintained in the proenzyme form because the prodomain occludes the catalytic center of the enzyme while covalently tethered to the rest of the molecule, and in so doing, it masks some IgE epitopes. This results in proteolytically latent HDM allergens being less immunogenic than their catalytically competent mature forms (Takai et al., 2005). Differences in prodomain structure between the HDM allergens and cathepsins have functional implications which place them in an identifiable subfamily of C1 enzymes (Zhang et al., 2007). The group 1 allergen prodomains from *Dermatophagoides* species comprise 80 amino acids arranged as four *α*-helices. Thus, they can be distinguished from cathepsins K, L, and S, in which the longer propieces comprise only three *α*-helices and contain a conserved “ERFNIN” motif [EX_3_RX_2_ (I/V)FX_2_NX_3_IX_3_N] that is absent in the HDM allergens (Zhang et al., 2007). Other cathepsins, notably cathepsin B, have prodomains which lack a further *α*-helix and which are shorter than the HDM allergens. In cathepsins, untethered propeptide units generally behave as regulators of their cognate proteases, but this seems not the case in Der p 1 (and, by inference, other group 1 HDM allergens) where maturation of the protease results in rapid degradation of the propiece and unconstrained enzymatic activity (Zhang et al., 2007). The corresponding group 1 allergen Blo t 1 of *B. tropicalis*, found only in tropical regions, represents an intermediate case between the more pervasive *Dermatophagoides* group 1 allergens and cathepsins. At the time of writing, catalytic competence of Blo t 1 is only inferred from alignment of its putative catalytic residues (C^119^ H^263^ N^283^) with Der p 1, and formal proof of the enzymatic activity of unambiguously pure allergen is awaited. It differs from its *Dermatophagoides* counterparts in sharing only ∼30% sequence identity overall and having a modified ERFNIN motif (ERFQVN), which may indicate that its prodomain could remain a potential inhibitor after maturation, restricting its behavior. In that regard, it is noteworthy that although Blo t 1 is clearly a major allergen of *B. tropicalis*, it is not obviously serodominant, unlike the group 1 HDM allergens. It is tempting to speculate that the lack of serodominance may be related to inferences that can be drawn about control of its proteolytic capacity.

Of numerous difficulties encountered in studying the biology of allergens, a fundamental challenge is their purification from the complex mixture of bioactive materials with which they are normally associated. Although such extraneous materials may have deep relevance to host immune responses and disease development, they can be serious confounding factors when investigating functions and mechanisms at the molecular level. A pertinent example was the claim, which has enjoyed a persistence in some literature, that Der p 1 is a multifunctional protease with both cysteine and serine protease activities (Hewitt et al., 1997). It is now clear that this is incorrect: no evidence for this notion exists within the crystal structure of Der p 1, and studies with native and recombinant Der p 1 in the presence of highly selective inhibitors provide no functional support. The likeliest explanation for the misleading claim is that the native Der p 1 preparation studied by those researchers was contaminated with serine peptidase allergens because native Der p 1 prepared by a strategy to rigorously eliminate such contamination fails to degrade the chymotrypsin substrate *N*-succinyl-AAPF-*p*-nitroanilide. In contrast, both trypsin and Der p 1 degrade *N*-Bz-FVR-*p*-nitroanilide, but whereas Der p 1 is inhibited by (S)-3-((S)-2-((S)-2-benzamido-3-methylbutanamido)propanamido)-2-oxoheptyl-2,6-bis(trifluoromethyl)benzoate (ADZ 50,000), it is not affected by a generic serine peptidase inhibitor, aminoethylbenzenesulfonyl fluoride, which blocks the action of trypsin (our unpublished data).

The cleavage specificity of Der p 1 has been evaluated in peptide sequences from suspected target proteins and by library screening campaigns. Substrates in a combinatorial library of general formula Abz-1-2-3-4-Y(NO_2_)-D-NH_2_, with cleavage between residues 3 and 4, revealed the following preferences for Der p 1: 1) V>A,Q,L,F; 2) A>>Q or K; 3) L, J, or A>S; and 4) S. Noting that J is an isostere of K, these findings are similar to the results of screening crude HDM extracts in a different combinatorial library where the preferences for the S_1_-S_4_ pockets were K, A, V, and J, respectively. A more recent campaign using phage display has proposed a preference for K/R,V,L/V,V,V for the S_1_-S_5_ pockets and used a bioinformatics approach to predict potential cellular substrates, some of which have not previously been revealed by hypothesis-driven investigation (Jacquet et al., 2017). Although informative about the selectivity or promiscuity of enzyme pockets, this information does not, of course, translate directly into obvious function or provide immediate chemical design solutions for inhibitor discovery. Potential substrates may be clandestine in cellular reality and, therefore, of marginal biologic significance. Conversely, apparently disfavored substrates may be presented in ways which encourage attack or whose cleavage initiates amplification mechanisms which transform the wider biologic significance of the target. For inhibitor design, an appreciation of the binding site topology taken together with substrate preferences is merely a first step in the creation of developable chemical entities as opposed to tool compounds whose value is restricted.

Examination of the structure of Der p 1 determined from crystals of isoallergen Der p 1.0105 reveals the presence of three disulphide bridges (C^84^-C^197^, C^111^-C^151^, and C^145^-C^183^) and suggests that the amino acid residues surrounding the substrate-binding groove, with its characteristic catalytic triad of C^114^, H^250^, and N^270^, are well conserved. The clear implication is that substrate preferences are consistent among isoallergens in a single species of HDM and between homologous allergens in different HDM species. This is supported by enzyme kinetic and inhibitor data (our unpublished data) and structural comparison of the active sites of Der p 1 and Der f 1, where substitution of R^231^ in Der p 1 by Q^232^ is the most notable difference.

High-resolution structural data allow the binding pockets of Der p 1 to be compared with off-target enzymes which have the potential to constitute selectivity nuisances in drug design. An empirical observation made in early discovery research was that nonoptimized inhibitors of Der p 1 were effective inhibitors of cathepsin B but showed good selectivity over cathepsins S and K. We believe that the selectivity of our initial inhibitors over cathepsins S and K was imparted to some extent by a small P_2_ group, whereas cathepsins S and K prefer larger groups in this position. Despite cathepsin B being the least like Der p 1 when judged by amino acid sequence, optimization of selectivity over cathepsin B requires more chemical design effort to exploit the greater openness of the S_3_ pocket in Der p 1 which results from substitution by T^74^ in place of the bulkier Y^75^ in cathepsin B. The appreciation of these differences provided encouragement that novel inhibitors of the HDM allergens with appropriate selectivity profiles were achievable.

While not compromising the view that they are a single target for structure-based protease inhibitor design, detailed crystallographic investigations have revealed some minor differences between Der p 1 and Der f 1. Examples include the presence of a binding site for divalent cations which may be occupied in the case of Der p 1, but not Der f 1 (Meno et al., 2005, 2006; de Halleux et al., 2006; Chruszcz et al., 2009). Removal of divalent cations does not alter the proteolytic activity of Der p 1 (unpublished data), which distinguishes it from papain in which divalent cations increase catalytic activity (Chruszcz et al., 2009). A further distinction between Der p 1 and other cysteine peptidases is its tendency to form oligomers in solution or in crystalline solid state (de Halleux et al., 2006; Chruszcz et al., 2009), although Der f 1 lacks this behavior (Chruszcz et al., 2009). Whether this oligomerization has biologic significance is unknown—the property is uncommon in cysteine peptidases generally, with cathepsin C being one of the few enzymes where it may be physiologically relevant. Although the IgE-binding epitopes of these allergens remain to be fully defined, structural analysis of a monoclonal antibody which binds both Der p 1 or Der f 1 shows that mutagenesis of the conserved allergen-binding site diminishes IgE binding, providing insight into a surface region which provides linear and/or conformational features for species cross-reactivity between Der p 1, Der f 1, and *E. maynei* 1 (Chruszcz et al., 2012). This region is distant from the catalytic sites of these allergens, suggesting that IgE-bound allergens are catalytically active, creating the potential for the exercise of multiple bioactivities when interacting with sentinels such as mast cells and basophils. A further feature of this site is its poor conservation in Blo t 1, consistent with the low cross-reactivity of *Blomia* and *Dermatophagoides* allergens in allergic sera (Chruszcz et al., 2012).

## Allergy Initiators: Biologic Effects of Group 1 HDM Allergens

For any protein to merit interest as a drug design target, a minimum requirement is that plausible evidence must exist to validate its association with a disease-related pathway. For group 1 HDM allergens, a clear association of the allergen with disease through precedented (i.e., IgE-dependent) mechanisms is historical reality, and persuasive contemporary evidence now links proteolytic activity to mechanisms considered core to the pathogenesis of allergy. These are summarized in Table 2 and examined in depth in the subsequent sections. For these reasons, the group 1 HDM allergens may be considered “initiator” allergens, a highly exclusive cadre whose actions underpin and facilitate progressive polysensitization to allergens from unrelated sources (although the number of people exhibiting monosensitization is also significant). It is clear from Tables 1 and 2 that the HDM allergen repertoire contains three serine proteases which replicate some of the effects of the group 1 cysteine proteases. In the airways, the nature of this redundancy underscores the selection of group 1 HDM allergens as the favored target, because the latter enable the redundancy of the serine peptidases through their ability to inactivate airway serpins while evading antiprotease defenses themselves. This provides a basis to anticipate that inhibition of a group 1 allergen will boost the defense against serine peptidase allergens by serpins. As discussed later, the correct proposal of protease target is endorsed by encouraging data for group 1 inhibitors in a variety of mechanistic models.

**TABLE 2:** Allergy-related biologic actions and effects evoked by HDM protease allergens

Sphere of Action	Action or Effect	Allergen or Extract	References
Mucosal defense	Cleavage of tight junctions/reduced epithelial barrier function/allergen delivery	HDM fecal pellet extract, Der p 1, Der f 1, Der p 3, Der p 6	Herbert et al. (1990, 1995), Winton et al. (1998), Wan et al. (1999, 2000, 2001), Nakamura et al. (2006); ADI program—data on file
	Disruption of epithelial adherens junctions	Der p 1 (directly and indirectly via ADAM 10)	Wan et al. (1999); ADI program—data on file
	Inactivation of airway antiproteases	Der p 1	Kalsheker et al. (1996), Brown et al. (2003)
	Epithelial-mesenchymal transition	HDM extract	Heijink et al. (2010a), Frisella et al. (2011)
	Inactivation of surfactant proteins	Der p 1, Der f 1	Deb et al. (2007)
	IgE-independent mast cell stimulation	Der p 1, HDM extract	Machado et al. (1996)
	Activation of NLRP3 inflammasome and apoptosis	HDM extract, Der p 1	Winton et al. (1998), Baker et al. (2003), Frisella et al. (2011)
Cell signaling	Prothrombinase activity	HDM fecal pellet extract, Der p 1	Zhang et al. (2016, 2018)
	PAR-1 and PAR-4 activation	Der p 1 (indirectly as prothrombinase)	Zhang et al. (2016); ADI program—data on file
	PAR-2 activation	HDM fecal pellet extract, Der p 1, Der p 3, Der p 9	Sun et al. (2001), Asokananthan et al. (2002), Jeong et al. (2008), Kato et al. (2009), Cho et al. (2012), de Boer et al. (2014), Post et al. (2014), Zhang et al. (2016), Reddy and Lerner (2017)
	PAR-1 inactivation	Der p 1	Asokananthan et al. (2002)
	PAR-2 inactivation	Der p 1	Adam et al. (2006), Kato et al. (2009)
	PAR-1 and PAR-2 upregulated expression	Der p 1	Shi et al. (2010)
	Mas-related G-protein–coupled receptor activation	Der p 1	Reddy and Lerner (2017)
	EGFR activation in epithelial cells	Der p 1 (indirectly as prothrombinase)	Zhang et al. (2016, 2018); ADI program—data on file
	Pannexon gating in epithelial cells	Der p 1 (indirectly as prothrombinase)	Zhang et al. (2016, 2018); ADI program—data on file
	ATP release from epithelial cells	Der p 1 (indirectly as prothrombinase)	Zhang et al. (2016, 2018); ADI program—data on file
	ADAM 17 activation in epithelial cells	Der p 1 (indirectly as prothrombinase)	Chen et al. (2017), Zhang et al. (2018)
	ADAM 10 activation in epithelial cells	Der p 1 (indirectly as prothrombinase)	Chen et al. (2017); ADI program—data on file
	ADAM 8 activation in epithelial cells	Der p 1 (indirectly as prothrombinase)	ADI program—data on file
	Cleavage of low-affinity IgE receptor (CD23)	Der p 1 (directly and indirectly via ADAM 10)	Schulz et al. (1995); ADI program—data on file
	Activation of endogenous interstitial prothrombinase	HDM extract (due to Der p 1)	Zhang et al. (2016); ADI program—data on file
Regulation of gene expression and exploitation of antioxidant deficits	ROS generation	Intracellular ROS by HDM fecal pellet extract, Der p 1 in epithelial cells, Der f 1 in neutrophils	Fukunaga et al. (2011), Zhang et al. (2016, 2018), Chen et al. (2017)
Immune activation and allergic polarization	Breaking of immune tolerance	HDM extract, Der p 1	Gough et al. (1999, 2001, 2003); ADI program—data on file
	Suppression of indoleamine 2,3-dioxygenase	Der p 1	Maneechotesuwan et al. (2009)
	TLR4 ligation	HDM extract, Der p 1 (indirectly as prothrombinase)	Zhang et al. (2018); ADI program—data on file
	Bioactivating cleavage of IL-33	Der p 1	Cayrol et al. (2018)
	Cytokine/chemokine expression and release (e.g., IL-33, TSLP, CCL2, CCL20, GM-CSF, IL-8, IL-13, etc.)	HDM extract, Der p 1, Der p 3, Der p 6, Der p 9	King et al. (1998), Pichavant et al. (2005), Adam et al. (2006), Kauffman et al. (2006), Ogawa et al. (2008), Kato et al. (2009), Shi et al. (2010), Arlian and Morgan (2011); ADI program—data on file
	Cleavage of IL-2R (CD25)	Der p 1	Schulz et al. (1998)
Antigen-presenting cell recruitment and activation	Dendritic antigen-presenting cell recruitment	HDM extract, Der p 1	Pichavant et al. (2005), Robinson et al. (2012); ADI Program—data on file
	Cleavage of DC-SIGN/DC-SIGNR	Der p 1	Furmonaviciene et al. (2007)
Interaction with viral RNA sensors	Transductional convergence with signaling from TLR3 and TLR7	HDM fecal pellet extract, Der p 1	Zhang et al. (2018); ADI Program—data on file
Effector mechanisms	Leukocyte recruitment	Der p 1, HDM extract	Robinson et al. (2012), Newton et al. (2014); ADI program—data on file
	Kinin generation from low- and high-molecular-weight kininogens	Der f 3	Takahashi et al. (1990), Maruo et al. (1991), Maruo et al. (1993)
	Decreased cysteine protease inhibitory activity of kininogens	Der f 3	Maruo et al. (1993)
	Anaphylatoxin generation	Der f 3	Maruo et al. (1997)
	IgE-independent “pseudoallergic” bronchoconstriction	Der p 1, HDM extract	ADI program—data on file

DC-SIGNR, DC-SIGN receptor; EGFR, epidermal growth factor receptor; GM-CSF, granulocyte macrophage-colony stimulating factor.

### 

#### Mucosal Defenses.

Antigen-presenting cell networks of the lung (dendritic cells) and skin (Langerhans cells) are the sentinels which link innate and adaptive immune responses. These cells process signals from activated innate immune mechanisms, such as pattern-recognition receptors, and present antigen to T cells after their migration to lymph nodes. To minimize nuisance triggering by low-grade external threats or the host microbiome, antigen-presenting cells and pattern-recognition receptors exhibit a polarized distribution, with many of these receptors and cells protected by the epithelium. Epithelial cells, airway macrophages, and components of airway surface liquid are thus the front line of defense against allergens and other immunologic threats. In understanding the initiation of sensitization and the processes which ensure its persistence, a key question is how allergens contact antigen-presenting cell networks, either by crossing mucosal barriers themselves or by promoting the ability of antigen-presenting cells to sample from the external environment. Of all allergens, the proteases of HDM have been the most studied in this regard, notably in the airways whose simple epithelial structure, intercellular junctions, and permeability characteristics are well characterized. The more complex stratified epithelium of skin is less extensively studied in the context of allergen disposition, reflecting that the role of tight junctions (TJs) in regulating skin permeability was itself unclear until the beginning of this millennium.

**Fig. 1.**
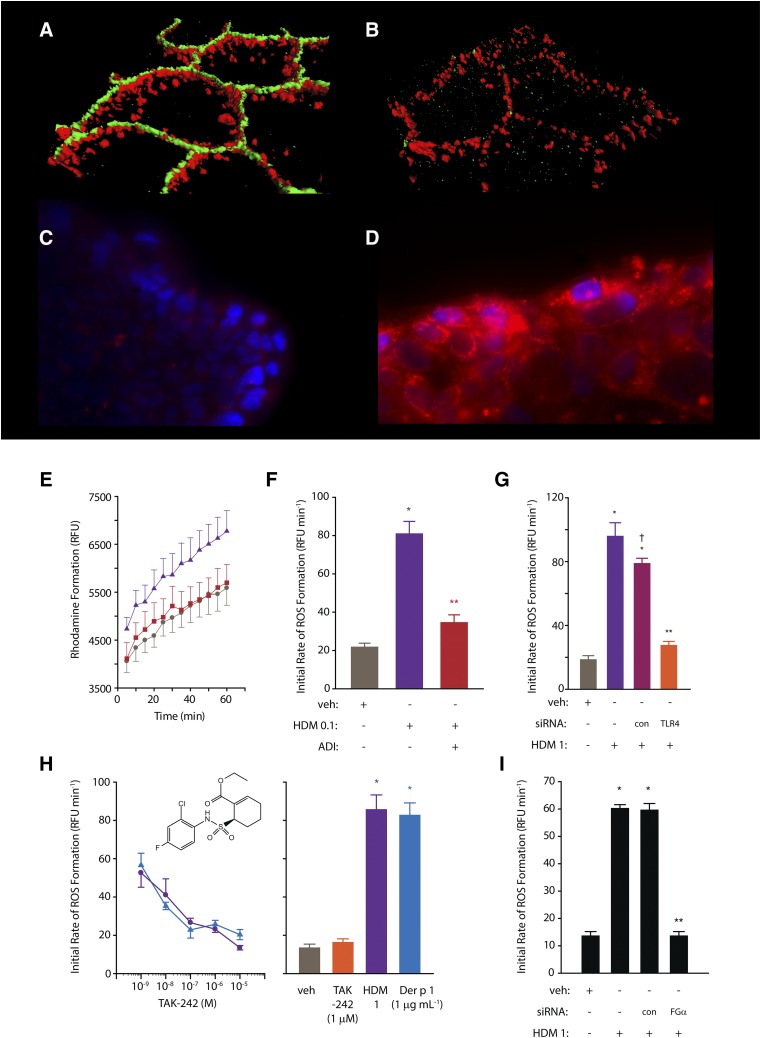
(A and B) Three-dimensional isosurface reconstruction of fluorescent antibody labeling of TJs (green) and desmosomes (red) in human airway epithelial cells. Normal cells are shown in (A); note the contiguous rings of TJs compared with the punctate staining of desmosomes. (B) Two hours after exposure to HDM allergen, note the loss of TJ staining, whereas desmosomes remain intact. (C and D) Human airway epithelial cells labeled with NucBlue and MitoSOX red (Life Technolgies, Renfrewshire, UK) in the absence of HDM allergen stimulation (C) or following exposure to mixed HDM allergens showing generation of intracellular ROS (D). (E) Progress curves showing formation of rhodamine in calu-3 cells loaded with dihydrorhodamine under control conditions (circles), after stimulation by mixed HDM allergens (triangles), or after allergen stimulation in the presence of an allergen delivery inhibitor (squares). (F) Initial rates of ROS formation derived from rhodamine formation in (E). (G) Silencing of TLR4 expression by siRNA attenuates intracellular ROS generation evoked by mixed HDM allergens in calu-3 cells. Data for transfected control cells (con) and nontransfected cells are shown for completeness. (H) Concentration-dependent inhibition of intracellular ROS formation by TAK-242 (an inhibitor of the association between TLR4 and the signaling adapter proteins TIRAP and TRAM) in calu-3 cells stimulated by mixed HDM allergens (circles) or Der p 1 (triangles). The bar chart depicts the rate of ROS formation (dihydrorhodamine oxidation) in unstimulated cells or in the presence of allergen activation. (I) Gene silencing of the production of the *α*-chain of fibrinogen inhibits ROS generation by mixed HDM allergens. For (E–H), all data are shown as the mean ± S.E. with *n* = 4–8. *P < 0.001 vs. vehicle (veh); **P < 0.001 vs. HDM; ^†^*P* < 0.05 vs. nontransfected HDM control. HDM 0.1, 1 refer to a natural mixture of allergens containing 0.1 or 1 *µ*g ml^−1^, respectively. Further methodological details concerning the studies in (C–I) are available (Zhang et al., 2016, 2018), and these form the basis of previously unpublished or recomposited data shown here. RFU, relative fluorescence units.

The first detailed studies of HDM allergen interactions with epithelial cells used a combination of functional, biochemical, and two-photon molecular excitation microscopy with three-dimensional isosurface image reconstruction, although earlier examination of permeability effects had been reported several years earlier (Herbert et al., 1990, 1995; Wan et al., 1999, 2000, 2001). These investigations revealed that TJs in human airway epithelial cells exposed to HDM fecal pellets were cleaved under conditions that mimic daily exposure to the allergens (Fig. 1), resulting in a nonspecific increase in paracellular permeability (i.e., the barrier becomes leaky to all allergens, regardless of origin, and may also facilitate dendritic, and other, cell migration). The underlying mechanism is a proteolytic attack by cysteine peptidase and serine peptidase HDM allergens on the extracellular domains of occludin and claudins (Wan et al., 1999, 2001), transmembrane proteins which form the contiguous intercellular contacts at the apical pole of the cells and which are adhesive components of the supramolecular TJ complex. In turn, this extracellular proteolytic cleavage initiates intracellular processing of the TJ plaque protein, ZO-1. Whereas epithelial permeability control is likely to depend on the ensemble function of various TJ adhesion proteins, lung-specific claudin-18 is known to be important in the type 2 T-helper cell (Th2)–high asthma phenotype. In common with claudins of TJs in other tissues, claudin-18 is downregulated by IL-13 (whose release is augmented by peptidase allergens), and its loss impairs epithelial permeability control in model systems in vitro and in vivo (Sweerus et al., 2017). Thus, the ability of protease allergens to cleave TJs directly and to affect their function through cytokine expression provides a powerful mechanism to compromise epithelial defense. As described later, this is accompanied by protease-driven inflammatory events in which the intracellular generation of reactive signaling molecules plays a key role and which provides a link to the activation of Toll-like receptor 4 (Fig. 1; Table 2).

An analogous mechanism operates in skin where HDM protease allergens cause the epidermis to become leaky and then impede its restitution (Nakamura et al., 2006; Jeong et al., 2008), while at the same time promoting cytokine and chemokine production, which is similar to the response in airways (Arlian et al., 2008; Ogawa et al., 2008; Oshio et al., 2009; Arlian and Morgan, 2011) (Table 2). The cleavage of TJs in keratinocytes affords the opportunity for Langerhans cells, which express TJ proteins themselves, to form new TJs with keratinocytes, enabling an upregulation of antigen sampling while, at least initially, retaining barrier integrity (Kubo et al., 2009). Similar behavior occurs in allergic rhinitis where, unlike nonrhinitic controls, the dendrites of antigen-presenting cells penetrate beyond the apical surface of the nasal epithelium (Takano et al., 2005). However, chronic allergen exposure or other predisposing factors for epithelial leakiness, such as loss of function mutations in filaggrin or dysregulation of lipid composition in the stratum corneum of the skin, result in augmented immunologic responsiveness to allergens. Indeed, emerging evidence suggests that clinically unaffected skin barrier properties in atopic dermatitis are compromised, as decreased levels of claudins-1, -4, and -23 have been found in nonlesional skin, and an inverse relationship between claudin-1 and Th2-polarized responses has been observed (De Benedetto et al., 2011; Brandner et al., 2015).

Although the allergen repertoire of HDMs consist of several proteases, the particular importance of group 1 allergens, such as Der p 1, is indicated by the prevention of transepithelial delivery of allergen when TJ cleavage is blocked (Wan et al., 1999), and when the proteolytic activity of Der p 1 is inhibited, intranasally administered HDM allergens no longer evoke allergic sensitization in mice. Thus, the serine protease HDM allergens, despite cleaving TJs (Wan et al., 2001), have a subordinate role in driving allergic sensitization.

In addition to the physical defenses, epithelial surfaces offer protection against pathogens through biochemical mechanisms (Table 2). Antiproteases are one facet of biochemical defense relevant to interactions with protease allergens, especially in the airways where epithelial surface liquid is rich in *α*_1_-antitrypsin capable of inhibiting the effects of serine protease allergens. However, Der p 1 inactivates this serpin and is itself resistant to most mammalian antipeptidases except *α*_2_-macroglobulin, whose molecular weight restricts its presentation at the airway surface (Kalsheker et al., 1996; Brown et al., 2003). Other targets of Der p 1 in airway surface liquid are surfactant proteins –A and –D, members of the collectin family of C-type lectin receptors. The function of these proteins in innate immunity is opsonization of pathogens for phagocytosis, but as their names imply, they also control surfactant production and may offer some protection against allergy development (Deb et al., 2007).

#### Cell Signaling and HDM Protease Allergens.

In addition to the events that promote the physical interaction of allergens with antigen-presenting cells, HDM protease allergens initiate a sophisticated series of events within the sphere of innate immunity. Cells that are in the front line of interactions with allergens (viz. macrophages, epithelial cells, keratinocytes, dendritic cells, and mast cells) express an extensive palette of innate immune receptors which are central to the events that initiate and perpetuate allergy. Cytokine production is one of their outputs with a prominent role in progressively driving disease symptoms and exacerbations. The main goal of clinical management in allergy is prevention of exacerbations, so interruption of this chain of events at key checkpoints should offer significant patient benefit.

#### Group 1 HDM Allergens Are Prothrombinases.

**Fig. 2.**
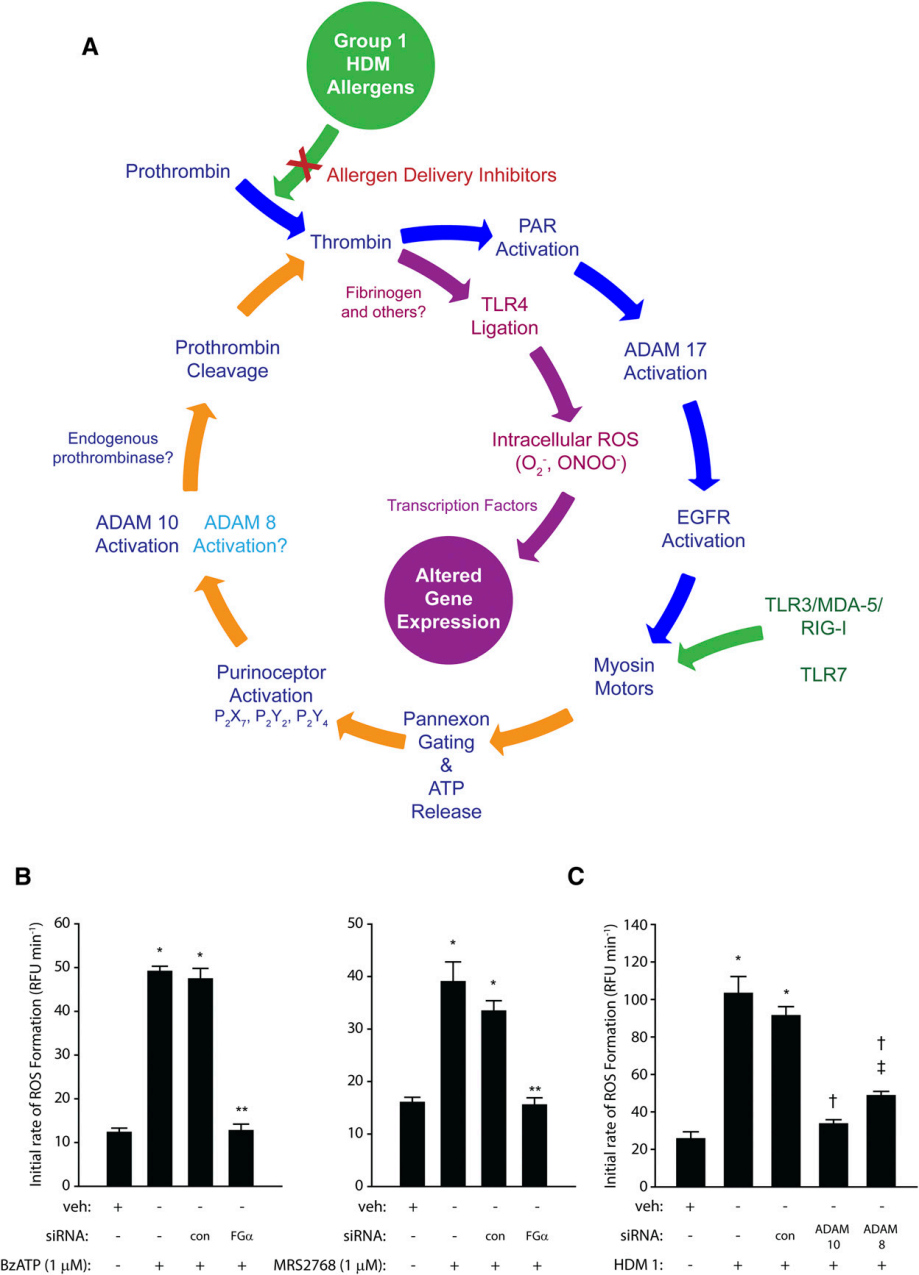
(A) The prothrombinase activity of Group 1 HDM allergens stimulates intracellular ROS formation in airway epithelial cells through an ATP and ADAM 10–dependent regenerating cycle initiated by the canonical cleavage of PAR-1 and PAR-4. The formation of endogenous ligands for TLR4 plays a key permissive role in this process because silencing of receptor expression or prevention of TLR4 interactions with intracellular adapter proteins blunts ROS formation. This signaling cycle receives convergent stimulatory inputs from viral RNA sensors (TLR3/MDA-5/RIG-I/TLR7) upstream from the gating of pannexons by myosin motors. ROS provide transcriptional regulation of gene expression through multiple mechanisms. Direct formation of thrombin by group 1 HDM allergens (prothrombinases) appears to be functionally compartmentalized from the ADAM 10–dependent generation of prothrombin because PAR-1, PAR-4, and pannexin-1 are required for the former. Although the underlying reasons for this dichotomy are not established, it may reflect poor accessibility of apically generated ligands to TLR4 in a polarized epithelium. The figure was revised and updated with added mechanistic details from the scheme originally published by Zhang et al. (2018). (B) Gene silencing production of the *α*-chain of fibrinogen in calu-3 airway epithelial cells inhibits ROS generation activated by ligation of P_2_X_7_ (2′(3′)-O-(4-benzoylbenzoyl)adenosine 5′-triphosphate, BzATP) or P_2_Y_2_ (uridine-5′-tetraphosphate δ-phenyl ester tetrasodium salt, MRS2768) receptors. *P < 0.001 vs. vehicle (veh) controls; **P < 0.001 vs. stimulated cells (nontransfected and transfection control, con). (C) Gene silencing of ADAM 10 or ADAM 8 attenuates ROS production by calu-3 cells stimulated by mixed HDM allergens containing Der p 1 at 1 *µ*g ml^−1^. *P < 0.001 vs. veh controls; ^†^*P* < 0.001 vs. stimulated cells; ^‡^*P* < 0.001 vs. veh controls. Data in (B and C) are shown as the mean ± S.E. (*n* = 8). The new data shown in (B and C) used methods which have been published elsewhere (Zhang et al., 2016, 2018). EGFR, epidermal growth factor receptor; RFU, relative fluorescence units.

Unexpectedly, one of the proteolytic features of Der p 1 is its ability to behave as a prothrombinase, forming thrombin from prothrombin independently from classic coagulation pathways (Zhang et al., 2016) (Table 2). In airway epithelial cells, this effect of Der p 1 is augmented by the subsequent activation of an endogenous prothrombinase, which plays a major role in controlling the generation of reactive signaling molecules (Zhang et al., 2018). These recent discoveries cast an exciting new perspective on how group 1 HDM allergens upregulate cytokine expression through the operation of a signaling cycle which interlinks components having a pleiotropic role in allergy specifically, or innate immunity generally (Fig. 2A). At the time of writing, the exact relationships between many key events in this cycle remain to be defined, but what is known suggests that group 1 HDM allergens are, by means of their proteolytic activity, the ignition keys of fundamental innate responses. Consequently, novel molecular entities which inhibit the proteolytic activity of group 1 HDM allergens affect a broad spectrum of events relevant to allergy initiation and maintenance (Zhang et al., 2018).

Through its interstitial prothrombinase activity, Der p 1 enables the canonical activation of protease-activated receptor-1 (PAR-1) and PAR-4 by thrombin in a manner consistent with the formation of a ternary complex of thrombin with receptor hetero-oligomers (Chen et al., 2017) (Table 2). This is the beginning of a regenerative cycle (Fig. 2A) from which key outputs are reactive oxygen and nitrogen species. Although natural mixtures of HDM allergens contain both serine and cysteine proteases, with the former as candidates for the activation of PAR-2, potent and selective inhibitors of Der p 1 attenuate the production of virtually all of the reactive species, suggesting that it is the major component within the repertoire of the allergen mixture responsible for their generation. That PAR-2 cleavage makes only a minor contribution is indicated by the small effects of either its pharmacologic antagonism or silencing by small interfering RNA (siRNA). Probe studies with a DNA-binding triphenylphosphonium analog of dihydroethidium indicate that at least some of this generation occurs in mitochondria through a two-electron reduction of oxygen, and this establishes the basis for a mechanism for the upregulation of cytokine production through well characterized mechanisms, notably those dependent on the transcription factor nuclear factor *κ*B (Zhang et al., 2016; Chen et al., 2017). Reactive oxidant species activate transcription factors and induce histone modifications favoring the induction of proallergic cytokines, whereas through direct transformation of proteins, they activate mitogen-activated protein kinase and the signal transducer and activation of transcription pathways which are implicated in allergy and asthma (Comhair and Erzurum, 2010; van Rijt et al., 2017). Other studies have provided direct evidence of DNA damage resulting from HDM exposure in mice and human lung cells, mirroring underlying events observed in asthma (Chan et al., 2016).

#### Group 1 HDM Allergens, Pannexon Gating, and ATP Release.

Downstream from the Der p 1–dependent activation of PAR-1/4 lies the opening of pannexons which, inter alia, result in the extracellular release of ATP (Fig. 2A; Table 2). These pannexons are assembled from pannexin-1, and the release of ATP operates as an innate alarm mechanism by signaling through P_2_X_7_, P_2_Y_2_, and, to a lesser extent, P_2_Y_4_ receptors, leading to the activation of A disintegrin and metalloprotease 10 (ADAM 10) and eventuating in the production of reactive oxygen species (ROS). The recruitment of ADAM 10 into this cycle is notable in that its other actions—namely, promoting IgE synthesis through its cluster of differentiation 23 (CD23) sheddase activity on B-lymphocytes (also a direct effect of Der p 1), and recruiting dendritic cells, eosinophils, neutrophils, and T cells through C-C chemokine ligand 20 (CCL20), CCL2, CCL5, C-X-C motif chemokine ligand 8 (CXCL8), and CXCL16 release—signify a pleiotropic role in allergy, whereas its sheddase action on E-cadherin of adherens junctions suggests an augmenting role in dysregulation of epithelial barrier permeability (Schulz et al., 1995; Gough et al., 2004; Weskamp et al., 2006; Inoshima et al., 2011; Mathews et al., 2011; Post et al., 2015) and the promotion of epithelial-mesenchymal transition (EMT). However, in airway epithelial cells responding to HDM allergens, it also appears that ADAM 10 initiates further conversion of prothrombin to thrombin—indirectly, directly, or both—but via events that are significantly downstream from the prothrombinase activity of Der p 1. The ligand shedding activity of ADAM 10 is Ca^2+^-dependent (Nagano et al., 2004; Reiss and Saftig, 2009), so an association with the opening of pannexons, which are permeable to both mono- and divalent cations, may be critical in its activation. Recent studies from our laboratory indicate that ADAM 8 may provide some support for ADAM 10 in this cycle, suggesting an analogous relationship to that involved in CD23 shedding, where ADAM 10 is the constitutive sheddase whose action can be duplicated, if necessary, by ADAM 8. The endogenous production of thrombin activated by ADAM 10 and ADAM 8 is required for the generation of reactive signaling species, which led us to consider what the further downstream consequences of these reactions might be.

#### Group 1 HDM Allergens and Toll-Like Receptor 4 Ligation.

Revealingly, we found that effective blockade of ROS formation occurred by siRNA silencing of Toll-like receptor 4 (TLR4) or pharmacologic inhibition of its interaction with the adapter proteins TIRAP and TRAM by ethyl-(6*R*)-6-(*N*-(2-chloro-4-fluorophenyl)sulfamoyl)cyclohex-1-ene-1-carboxylate (TAK-242) (Zhang et al., 2018). Attenuation of responses by a Der p 1 inhibitor shows that this activation of TLR4 requires Der p 1 and is independent of ligation by bacterial lipopolysaccharides (LPS), the archetypal agonists of this receptor (Zhang et al., 2018). Although LPS is present in house dust and HDM allergen mixtures, LPS is only weakly effective in triggering ROS production, at least in healthy airways, because of a deficiency in the myeloid differentiation protein-2 (MD-2) co-receptor and the polarized basolateral distribution of TLR4, which protects it from nuisance triggering by exogenous signals. However, TLR4 is activated by a range of putative endogenous ligands which include fibrinogen and its cleavage products (Hodgkinson et al., 2008; Erridge, 2010; Yu et al., 2010; Millien et al., 2013; Cho et al., 2017). Airway epithelial cells secrete fibrinogen from their basolateral aspect in a vectorial, microtubule-dependent manner and are one of few extrahepatic sites where all three component chains of the protein are expressed (Guadiz et al., 1997). This leads to the possibility that fibrinogen cleavage products are the agencies by which Der p 1–dependent TLR4 activation is achieved, although ensemble participation of other TLR4 ligands should not be excluded. The involvement of fibrinogen is indicated by attenuation of responses in airway epithelial cells in which expression of the FGA gene, which encodes the *α*-chain of the protein, has been silenced by siRNA (Fig. 2B). The Der p 1–dependent activation of TLR4 is, therefore, a consequence of the chain of signaling events initiated by its activation of PAR-1 and PAR-4, and the TLR4-dependent action of selective P_2_Y_2_R or P_2_X_7_R agonists suggests that the coupling of TLR4 ligation to ROS production occurs downstream from the gating of pannexons (Zhang et al., 2018).

Discovery of a linkage between Der p 1 and TLR4 is of considerable interest because TLR4 expressed on airway epithelial cells is an indispensable requirement for the development of allergic sensitization to HDM allergens (Hammad et al., 2009). Ligation of TLR4 leads to an activation of cells by IL-1*α* and the release of granulocyte macrophage colony-stimulating factor and IL-33 (Willart et al., 2012), the latter being a bioactivation target of the Der p 1 enzyme also (Cayrol et al., 2018) (Table 2). The importance of TLR4 and this triad of cytokines is demonstrated by depletion of the receptor or cytokine neutralization preventing allergic responses (Hammad et al., 2009; Willart et al., 2012). Until the discovery of how Der p 1 leads to TLR4 activation, the mechanisms offered to account for the indispensability of TLR4 were: 1) the presence in PAR-2 of a potential Toll/interleukin-1 receptor domain which might bind to myeloid differentiation primary response 88 (MyD88) in the TLR4 signaling complex, which regulates early nuclear factor *κ*B–dependent gene transcription (Barrett et al., 2009); and 2) the similarity between group 2 HDM allergens and MD-2, which functions as a co-receptor protein in the response to LPS (Eisenbarth et al., 2002; Trompette et al., 2009). A structural feature linking the group 2 HDM allergens with MD-2 is the presence of a large hydrophobic pocket capable of binding lipophilic ligands such as LPS. The attraction of this MD-2 mimicry by group 2 HDM allergens is that it might provide a means to compensate for a relative deficiency of MD-2 in airway epithelial cells and offers a mechanistic connection to LPS which is implicated in allergy development under certain conditions. However, MD-2 mimicry suffers limitations as a central initiator mechanism, not least in failing to accommodate the cellular distribution of TLR4 in a healthy-airway epithelium. Unlike group 1 HDM allergens, those of group 2 neither initiate ROS production nor have a direct effect on epithelial barrier properties when protease contamination is rigorously excluded. Indeed, studies on the transepithelial disposition of Der p 2 show that significant permeation occurs only in the presence of proteolytically active group 1 allergens and can be prevented by a novel Der p 1 inhibitor of high potency. Moreover, inhibition of Der p 1 proteolytic activity prevents allergic sensitization in mice (Gough et al., 1999, 2001; Zhang et al., 2009; Robinson et al., 2012), an effect which is hard to reconcile with the indispensability of TLR4 being solely reliant on MD-2 mimicry. Our data provide an alternate rationale where the decisive event is thrombin formation by the direct prothrombinase activity of group 1 HDM allergens which, in turn, facilitates the formation of endogenous TLR4 ligands in the airway epithelium through activation of an additional endogenous prothrombinase (Zhang et al., 2016, 2018). The identities of these endogenous TLR4 activators remain unclear, but gene silencing of any one of the three component chains of fibrinogen abrogates ROS production by Der p 1 in human airway epithelial cells, although other ligands appear to participate too (Fig. 2B and our unpublished data). Significantly, selective inhibitors of Der p 1 provide effective inhibition of all these events (Zhang et al., 2018). It is evident from the foregoing that the airway epithelium is the cellular host of a sophisticated signaling nexus which combines the physical delivery of allergen to antigen-presenting cells, and thereby T-lymphocytes, with the creation of a signaling environment which transduces the progression from innate to acquired immunity with an allergic polarization.

#### Signaling Convergence Between Group 1 HDM Allergens and Viral RNA Sensors.

A fascinating aspect of this HDM allergen–dependent route to TLR4 ligation and the production of reactive intermediates which regulate gene expression is that the signaling mechanisms converge with cellular responses initiated by ligation of the viral RNA sensors TLR3 and TLR7 (Fig. 2A; Table 2) (Zhang et al., 2016, 2018). The point of convergence lies upstream from the myosin motor−dependent gating of pannexons and ATP release (Zhang et al., 2018). In allergic asthma, disease exacerbations arise from interactions between allergens and respiratory viruses (principally rhinovirus, respiratory syncytial virus, and influenza), so the identification of a nexus linking these stimuli provides new insight into how these exacerbations are precipitated. Interestingly, given the regenerative cycle which underlies this production of ROS, activation of PAR-1 contributes to the pathogenicity of influenza A, and PAR-1 and TLR3 are both upregulated by infections caused by respiratory viruses (Groskreutz et al., 2006; Antoniak et al., 2013).

#### Thrombin and ATP: Innate Effector-Perpetuators in Allergy.

It has been known for some time that concentrations of thrombin in airway surface liquid are elevated in asthma to levels which are capable of driving cell proliferation, and they are also raised following respiratory virus infection (Terada et al., 2004). Whereas some thrombin may result from tissue-repair mechanisms activated by inflammation, more recent data suggest that it also functions as an innate strategic initiator and an effector-perpetuator of allergy through its direct generation by inhaled Der p 1 (Zhang et al., 2016). Similarly, ATP is present in elevated concentrations in bronchoalveolar lavage fluid in asthma (Müller et al., 2011), consistent with the allergen-dependent gating of pannexons. In addition to triggering ROS generation, it initiates the release of IL-33, which is pivotal in the orchestration of responses mediated by ILC2 cells and promotes a Th2 bias in dendritic cells (Idzko et al., 2007; Müller et al., 2011). The actions of ATP extend downstream from these events and additionally activate mast cells, eosinophils, and cause dyspnea (Schulman et al., 1999; Basoglu et al., 2005). In keeping with the foregoing, novel Der p 1 inhibitors which abrogate thrombin generation and pannexon-dependent ATP release also attenuate eosinophil recruitment, inhibit the release of IL-33 and TSLP, and impair acute allergic bronchoconstriction (Newton et al., 2014 and our unpublished data). Their ability to reduce IL-33 production in the airways removes a critical component of innate immune signaling which directs the development and persistence of allergic sensitization and some of its key pathophysiologic features.

#### Activation of PAR-2 and Mas-Related G-Protein–Coupled Receptor X1.

The discovery of PARs naturally prompted speculation that they could be receptors for protease allergens, especially those, like the group 3 and group 6 HDM allergens, with substrate preferences similar to canonical activators of these receptors. That group 1 HDM allergens would interact with one or more of these receptors was assumed to occur through an example of biased agonism. The ability of HDM allergen extracts and purified allergens to stimulate cytokine (e.g., IL-6, IL-8, CCL11, granulocyte macrophage colony-stimulating factor) release from airway epithelial cells or keratinocytes in a manner which paralleled the effects of PAR agonist peptides reinforced this view, especially highlighting PAR-2 as an important molecular recognition system for protease allergens with a central mechanism in disease (King et al., 1998; Kato et al., 2009). However, subsequent investigations suggested that an association of PAR-2 with asthma and other allergic conditions is complex. Illustratively, in mouse models of HDM sensitization, it is dispensable (Asokananthan et al., 2002; Adam et al., 2006; Post et al., 2014), and in some disease models, PAR-2 activation dampens rather than escalates inflammation (De Campo and Henry, 2005; Ebeling et al., 2005); in the case of group 1 HDM allergens, there are conflicting accounts of whether PAR-2 cleavage is an activation signal at all. Furthermore, the discovery that Der p 1 is a prothrombinase which initiates the PAR-1- and PAR-4–dependent ligation of TLR4 introduces a significant new dimension to how protease-mediated signaling promotes and maintains allergy.

An aspect of chronic asthma which may link Der p 1 and PAR-2 is airway remodeling and EMT, in which transforming growth factor-*β* is a key element (Hackett, 2012). Group 1 HDM allergens activate latent transforming growth factor-*β* and promote EMT characterized by a PAR-2 and epidermal growth factor receptor–dependent reduction of E-cadherin expression by epithelial cells (Nakamura et al., 2006; Heijink et al., 2010a,b; Frisella et al., 2011), which in turn is a stimulus for the upregulation of CCL17 and TSLP (Heijink et al., 2007). As described earlier, Der p 1 activates PAR-4, which itself is implicated as an initiator of EMT, and the ensuing cycle which leads to ROS formation involves ADAM 10, a key sheddase of E-cadherin (Ando et al., 2007; Inoshima et al., 2011; Chen et al., 2017).

Attention has also turned to the activation of other receptors in conjunction with PARs. Data have been obtained to show that Der p 1 activation of the orphan receptor Mas-related G-protein coupled receptor X1 (MRGPRX1) from the mas-related G-protein–coupled receptor family contributes to PAR-2–dependent cytokine release from cultured human airway epithelial cells (Reddy and Lerner, 2017), although the relevance to allergy remains untested. However, a possible link between Der p 1 and MRGPRs is intriguing because of their expression in sensory neurons and mast cells. The latter degranulate in response to the proteolytic action of Der p 1 through an uncharacterized mechanism independent from IgE cross-linkage (Machado et al., 1996). In the context of MRGPRs (and PARs) transducing the nociception of pain and itch, the ability of a protease to activate such physiologic responses is well established, not least through the use of spicules from pods of the cowhage plant (*Mucuna pruriens*), which contain the cysteine protease mucunain, as an experimental stimulus in human volunteers (Wolff and Goodell, 1952; Broadbent, 1953; Shelley and Arthur, 1955; Reddy et al., 2008). Others will be aware of mucunain's efficacy at inducing a burning pain by prank exposure to “itching powder,” a favorite product of novelty shops. One unexpected feature which became quickly evident in early studies with cowhage is that its ability to induce pruritis is heavily reliant on histamine release (Broadbent, 1953), suggesting that the concept of pseudoallergic, protease-dependent mast cell activation should be revisited in the context of contemporary receptor biology and innate immunity.

#### Group 1 HDM Allergens and Deficits in Antioxidant Defense.

One of the long-standing enigmas of allergy is why environmentally pervasive inhalant allergens evoke sensitization in only a subset of the exposed population. The generation of ROS by airway epithelial cells as an innate response to group 1 HDM allergens suggests a scheme to account for some of this difference in susceptibility. It is well established that a deficit in enzymatic and nonenzymatic antioxidant defenses is common in asthma (Sackesen et al., 2008; Comhair and Erzurum, 2010). Some of these are genetically determined (Fryer et al., 2000; Spiteri et al., 2000; Mapp et al., 2002), whereas others may arise consequentially from the development of disease. These deficits are exacerbated by the pathogenetic upregulation of oxidant production, such as the induction of NADPH oxidase subunits by cytokines. HDM allergen extract increases the selective expression of DUOX-1 in human airway epithelial cells, and this is correlated with enhanced release of hydrogen peroxide and IL-33 (Hristova et al., 2016). In asthma, allergen exposure increases DUOX-1 expression by the nasal epithelium, and neutrophil-derived ROS production by Der f 1 is greater than in controls without asthma (Fukunaga et al., 2011). The enhancement of ROS production by certain allergens may itself be a further factor in promoting the allergenicity of other exogenous proteins through carbonyl adduction to form reactive aldehydes which can direct a Th2 proliferative bias in T-lymphocytes (Moghaddam et al., 2011).

A key role for oxidant/antioxidant balance in shaping the development of allergy is suggested by compelling evidence from disease models. In mice, the cysteine protease papain promotes sensitization through mechanisms involving oxidative stress (Tang et al., 2010), and partial inhibition of protease activity blunts the capacity of HDM allergen extract to induce allergic inflammation (Utsch et al., 2015). Correspondingly, mice deficient in the master antioxidant regulator nuclear factor (erythroid-derived 2)-like 2 (Nrf2), or which are unable to upregulate it, develop enhanced responses to ovalbumin or HDM allergens (Rangasamy et al., 2005; Williams et al., 2008; Li et al., 2013; Utsch et al., 2015). Similarly, glutathione depletion exacerbates airway hyper-reactivity and inflammation (Nadeem et al., 2014). Consistent with this theme, an Nrf2 activator protected against IL-33 release and allergic responses (Uchida et al., 2017), whereas overexpression of Nrf2 enhanced the expression of ZO-1, occludin, and E-cadherin in the airway epithelium (Comhair et al., 2001).

#### Antigen-Presenting Cell Recruitment.

Accumulation of antigen-presenting cells at mucosal surfaces and their departure for interaction with T-lymphocytes is a strategic conduit linking innate and acquired immune responses to allergens. This is demonstrated by the observations that HDM allergen exposure recruits antigen-presenting cells to lung [dendritic cells (DCs)] and skin (Langerhans cells) through the agency of chemokine release (CCL2, CCL5, CCL20, and CXCL10) by epithelial cells (Pichavant et al., 2005), and that humanized SCID mice reconstituted with monocyte-derived DCs from patients with atopic asthma are predisposed to develop immune responses with a Th2 polarization (Hammad et al., 2002). Transcriptomic analysis in HDM allergen–exposed airway epithelial cells or clinical airway specimens suggests that chemokines directed against antigen-presenting cells constitute part of an upregulated core gene repertoire (Vroling et al., 2008a,b). Both CCL2 and CCL20 have been proposed as pivotal in the increased steady-state numbers of DCs in the bronchial mucosa of patients with asthma, with a notable increase in Der p 1–dependent CCL20 release in airway samples in atopic asthma (Pichavant et al., 2005). A role for CCL20 in response to HDM allergens is replicated in a mouse model where its release is TLR4-dependent (Hammad et al., 2009). CCL20 is a ligand for C-C chemokine receptor 6 (CCR6), which is expressed on Langerhans cell–like precursors of conventional DCs that are associated with inflammatory events. In mice deficient in CCR6, allergic pulmonary responses are attenuated due to impaired migration of DCs (Lukacs et al., 2001). In contrast to HDM allergen responses, signaling via the CCL20/CCR6 axis does not occur with ovalbumin (Robays et al., 2007), which requires the collateral priming effects of the proteolytic activity of Der p 1 or another adjuvant for the development of robust IgE responses (Gough et al., 1999, 2001; Fattouh et al., 2005; Zhang et al., 2009). Release of CCL20 from human airway epithelial cells by HDM allergen extracts also occurs through a *β*-glucan–dependent route. This mechanism is protease- and TLR4-independent but involves Syk activation, suggesting that it might be mediated through the C-type lectin receptor, dectin-1 (Nathan et al., 2009). Other work has suggested a role for *β*-glucan–dependent, TLR2-mediated signaling by HDM allergen extracts in the nasal mucosa (Ryu et al., 2013). Similar to chitins [*β*-(1,4)-poly-*N*-acetyl-d-glucosamine polymers] which also ligate dectin-1 (Lee et al., 2011), *β*-glucans are extraneous factors in HDM culture extracts and components, from multiple sources, of environmental house dust, so this activation of chemokine production by multiple mechanisms is anticipated as a multilayered reinforcement of allergic polarization. Although these observations suggest the importance of CCL20/CCR6 signaling in the allergic recruitment of DCs, the pattern of chemokine and receptor activation seems likely to be more complex given that the high phenotypic plasticity of antigen-presenting cells provides them with a versatile repertoire of delegated effector roles. For this reason, selective intervention at targets within this particular checkpoint in the initiation and maintenance of allergy may be of variable benefit compared with other options. Illustrating the difficulty in validating CCL20/CCR6 as a discrete, primary target pairing, CCL2, CCL5, and CXCL10 chiefly act via ligation of CCR1, CCR2, CCR5, and CXCR3 found on DCs of monocyte derivation, and of these combinations, the interaction of CCL2-CCL2R is particularly interesting in allergy development because CCR2^+^Ly6c^hi^ monocytes are precursors of inflammatory CD11b^+^ DCs found in allergy (Robays et al., 2007; Hammad et al., 2009).

Although antigen-presenting cell recruitment and activation may be driven primarily through signaling events triggered by HDM allergens in the airway epithelium, the allergens must also have direct interactions with antigen-presenting cells, and these have been the subject of some scrutiny in the context of protease-dependent activation and programming of Th2 polarization. One approach has been to use in silico prospecting for potential substrates of Der p 1 among the cell surface proteins found on dendritic cells, resulting in identification of C-type lectin receptor dendritic cell–specific intercellular adhesion molecule 3–grabbing nonintegrin (DC-SIGN) and its homolog DC-SIGN receptor as potential substrates of Der p 1 (Table 2). In support of this, recombinant DC-SIGN and DC-SIGN receptor are cleaved by Der p 1 in vitro (Furmonaviciene et al., 2007). DC-SIGN expression is reduced in DCs exposed to Der p 1, although it is hard to ascertain how much of this disappearance is due to proteolysis or to protease-independent endocytosis (Kauffman et al., 2006; Furmonaviciene et al., 2007).

The challenge of devising a successful antigen presentation checkpoint intervention is further demonstrated by another potential target of Der p 1 in DCs (Table 2). Der p 1 causes a downregulation of indoleamine 2,3-dioxygenase in HDM-sensitive individuals (Maneechotesuwan et al., 2009). Indoleamine 2,3-dioxygenase catalyzes the conversion of tryptophan to kynurenine and causes immunosuppression through a combination of tryptophan depletion (which activates a sequence of events through induction of general control nonrepressed 2 kinase) and the direct cellular effects of kynurenine and other tryptophan metabolites (Johnson et al., 2009). Consequently, downregulation of the enzyme by Der p 1 is a potentially interesting mechanism leading to the breaking of immune tolerance and the development and maintenance of sensitization. Indoleamine 2,3-dioxygenase activity is particularly important in antigen-presenting cells, although there is notable heterogeneity in its significance in different DC subsets, especially between conventional DCs and plasmacytoid DCs (Harden and Egilmez, 2012). The success of attempts to induce tolerance to an allergen by manipulation of indoleamine 2,3-dioxygenase activity may, therefore, depend on which subset(s) of DCs is most relevant. It should also be noted that chronically stimulating indoleamine 2,3-dioxygenase activity may not be generally beneficial because of the inherent risks associated with a concomitant attenuation of routine immune surveillance for pathogens and tumors (Muller et al., 2008; Harden and Egilmez, 2012). For these and other reasons, we preferred a different approach focused on the allergen rather than innate host checkpoints.

## Allergen Delivery Inhibitors

The strong causative association of serodominant group 1 HDM allergens with major allergic diseases makes them a compelling target in the search for unprecedented interventions intended to modify the basic immunologic events that are responsible for the development of disease. As outlined in the earlier sections, and summarized by the actions listed in Table 2, understanding how their proteolytic activity drives these sentinel events provides encouragement that inhibitors of this proteolytic activity offer the prospect of disease modification. The chemical design challenge associated with this unprecedented mechanism was significant, and the identification of developable candidates necessitated the rigorous pursuit of a clear vision of the required attributes, some of which would be deemed an unusual focus in most early-stage discovery research. Given the similarity of group 1 HDM allergens as proteases, this enabled us to use Der p 1 as the archetypal template for inhibitor design. We call this new class of drugs “allergen delivery inhibitors” to reflect that they interfere with the ability of HDM allergens to engage with antigen-presenting cells. As will now be evident, this process of “delivery” is a combination of biophysical events in intercellular junctions and the creation of a complex molecular signaling pathway which leads from innate immunity to the acquisition and persistence of allergic sensitization.

Our approach to Der p 1 inhibitor design was informed by the fact that the targets are inhalant allergens, and preventing their effects at the site of initial impaction in the lungs would be advantageous. With a nonhuman inhaled target, the interaction with an inhibitor has no requirement for cell permeation, which, in combination with properties designed to minimize systemic exposure, means a significant opportunity to mitigate safety risks in chronic therapy. There are several device options for the delivery of drugs by inhalation, viz. nebulizer, metered dose inhaler, and dry powder inhaler (DPI). We elected to pursue compounds that would be compatible with DPI delivery, with the expectation that this would bring confidence for usage in other devices. Delivery by DPI imposes a burden on chemical design and pharmaceutics because it demands nonhygroscopic, stable, crystalline compounds which can produce particles of consistent size that will distribute uniformly to the site of action in the lung, in addition to embodying the pharmacologic profile for target selectivity, potency, safety, and duration of action.

**Fig. 3.**
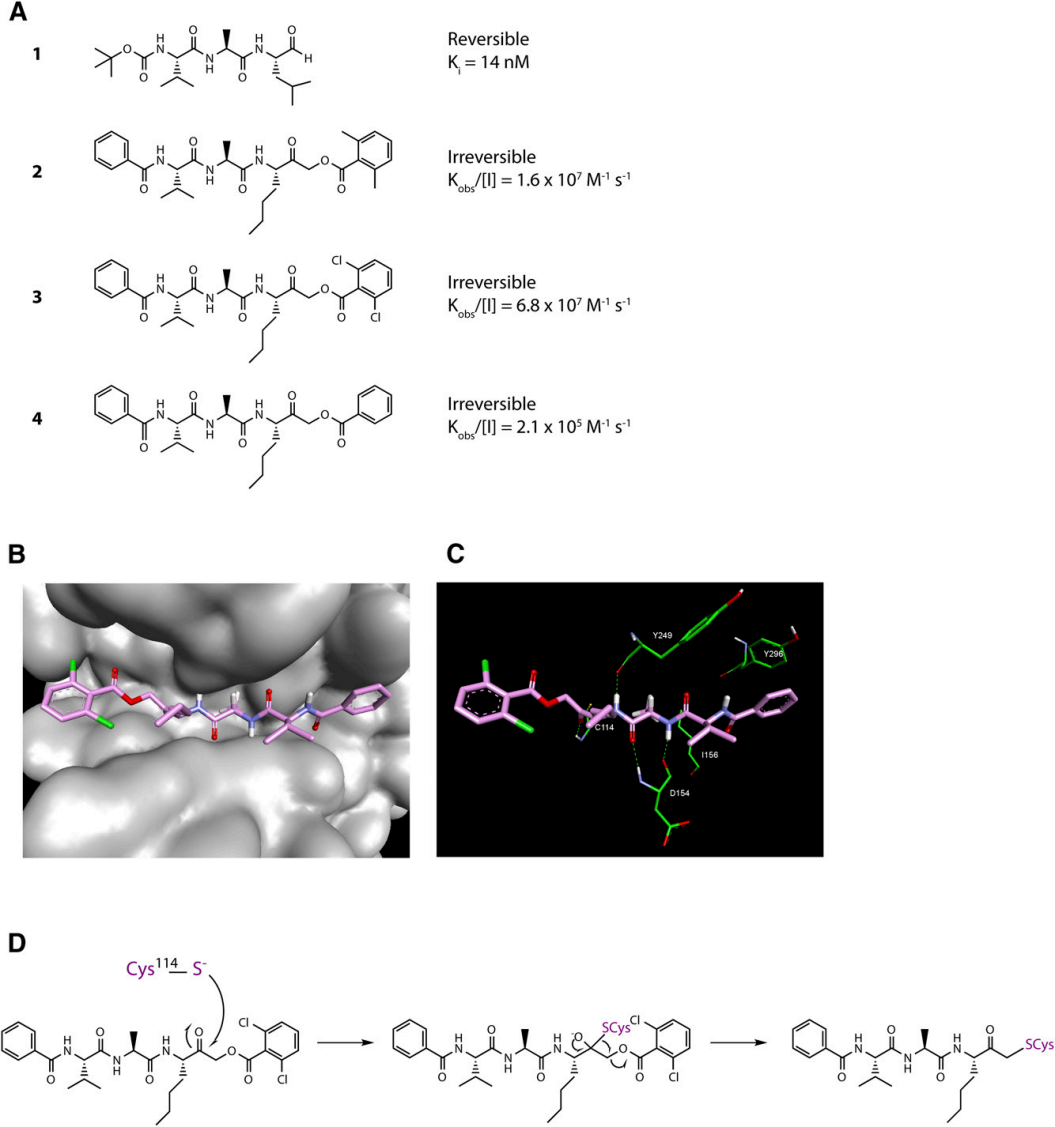
(A) Inhibitors of Der p 1 identified in early discovery research. Activity for the reversible inhibitor aldehyde **1** is expressed as the inhibition constant, whereas data for the acyloxymethyl ketones with irreversible action are expressed as second-order inhibitor rate constants. Measurements were performed as described by Newton et al. (2014) and the online supporting information, https://pubs.acs.org/doi/suppl/10.1021/jm501102h and https://pubs.acs.org/doi/suppl/10.1021/jm501102h/suppl_file/jm501102h_si_001.pdf, using (3S,6S,9S,12S,15S,18S)-1-(2-aminophenyl)-9-butyl-18-carbamoyl-15-(4-hydroxy-3-nitrobenzyl)-12-(hydroxymethyl)-3-isopropyl-6-methyl-1,4,7,10,13,16-hexaoxo-2,5,8,11,14,17-hexaazaicosan-20-oic acid as substrate. (B and C) Docking of compound **3** in the substrate-binding groove of Der p 1.0105 shown, respectively, as surface representation and simplified stick view revealing hydrogen bonding interactions with Derp 1. (D) General mechanism for irreversible inhibition of Der p 1 using acyloxymethyl ketone **3** as exemplar.

**Fig. 4.**
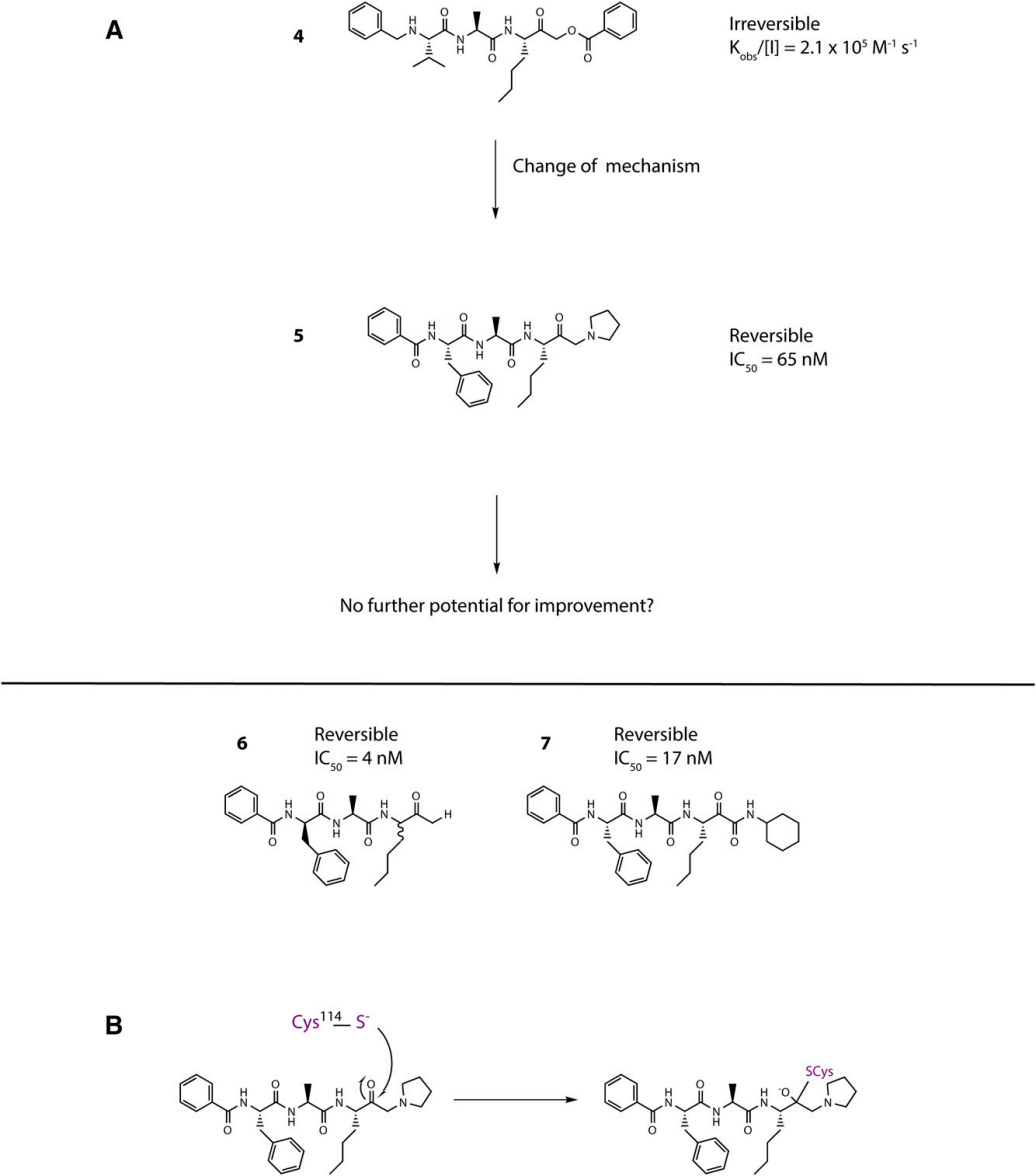
(A) Transition from irreversible to reversible binding mode inhibitors of Der p 1 by modification of the cysteine binding motif and identification of pyruvamide **7**. Inhibitor characteristics were determined as disclosed by Newton et al. (2014) and the online supporting information, https://pubs.acs.org/doi/suppl/10.1021/jm501102h and https://pubs.acs.org/doi/suppl/10.1021/jm501102h/suppl_file/jm501102h_si_001.pdf, using (3S,6S,9S,12S,15S,18S)-1-(2-aminophenyl)-9-butyl-18-carbamoyl-15-(4-hydroxy-3-nitrobenzyl)-12-(hydroxymethyl)-3-isopropyl-6-methyl-1,4,7,10,13,16-hexaoxo-2,5,8,11,14,17-hexaazaicosan-20-oic acid as substrate. (B) General mechanism for reversible inhibition of Der p 1 using amino ketone **5** as exemplar.

Opening explorations of Der p 1 inhibitor design were based around the introduction of inhibitory warheads into a modified peptide substrate sequence based on the scaffold peptide VAJS. Contrary to the development of compounds for oral delivery, it is usually advantageous for inhaled asthma drugs to have poor oral bioavailability to minimize systemic exposure and thus achieve a good safety profile. Therefore, compounds which violate Lipinski “rules” provide interesting freedoms for inhaled drug design which would not usually be tolerated in other settings. A peptide template, with a high proportion of hydrogen bond donors and acceptors in a flexible scaffold, was therefore considered a useful departure point for such an application. An exemplar derived from this scaffold is aldehyde **1** (Fig. 3), but despite encouraging potency, it was compromised by expectations of instability and nonspecific reactivity. Surprisingly, some warheads effective in the design of cysteine peptidase inhibitors directed at other targets were found to be poor inhibitors of Der p 1, such as vinylogous esters and sulfones elaborated from the scaffold of **1**. Initial progress in the search for potent Der p 1 inhibitors was made with the identification of a series of acyloxymethyl ketones exemplified by **2**, **3**, and **4** (Fig. 3). Docking of **3** in Der p 1.0105 (Protein DataBank: A2S8) suggested that the norleucine side chain makes hydrophobic interactions with the P_1_ pocket while the carbonyl initially forms a reversible covalent bond with C^114^, and the NH a hydrogen bond with Y^249^ (Newton et al., 2014). Consistent with the mechanism of inhibition for acyloxymethyl ketones, C^114^ subsequently migrates to displace the acyl group, forming an irreversible covalent bond with the inhibitor. The amino and carbonyl groups of alanine hydrogen bond with D^154^, and the preference for compact groups at P_2_ arises because of I^156^. The side chain of valine makes a hydrophobic interaction with the P_3_ pocket, and the benzoyl capping group forms hydrophobic *π*-stacking associations with tyrosine residues. Although docking analysis provided useful information to guide subsequent chemical design, the acyloxymethyl ketones, per se, lack developability, primarily through concerns about their irreversible inhibition of the target. Therefore, the challenge was to identify functional groups which would enable the creation of compounds whose target inhibition was reversible while maintaining the benefits of slow off-rate. To this end, we investigated a series of inhibitors which were capable of forming a reversible covalent bond with C^114^. An advance toward this goal was a series of amino ketones which demonstrated the achievability of incorporating a reversible inhibitor motif into a scaffold, which offered good prospects for optimization. However, despite initially encouraging progress, it proved impossible to increase potency beyond that of compound **5** (Fig. 4). This activity was deemed insufficient when rated against a compound developability profile whose potency requirements had been defined from empirical estimates of target exposure in humans and the need to be mindful of device dosage practicalities (Newton et al., 2014).

**Fig. 5.**
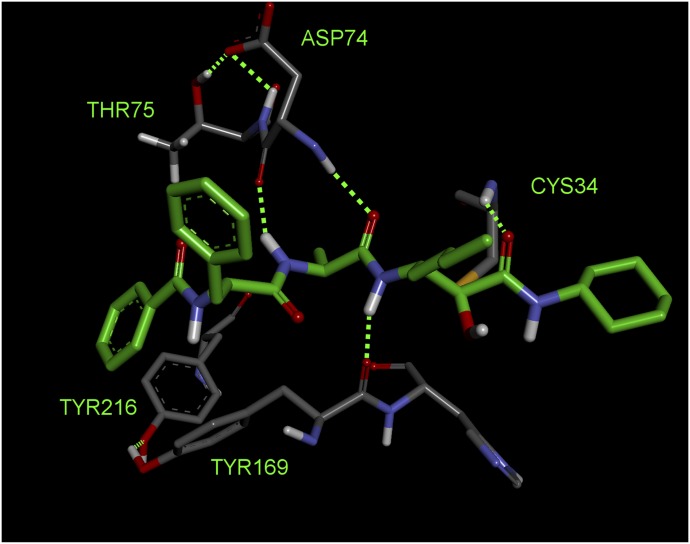
Docking model of compound **7** bound to the active site of Der p 1.0105. Procedural details are presented by Newton et al. (2014), https://pubs.acs.org/doi/suppl/10.1021/jm501102h.

Reassurance that an uplift in potency could be achieved in a reversible inhibitor was provided by **6** (Fig. 4), albeit using a functionality of limited developability. A pursuit of alternative groups led to the identification of a series of pyruvamides, such as **7** (Fig. 4), which became the focus for detailed exploration (Newton et al., 2014). The inhibitory potency of **7** against the Der p 1 target is compatible with the declared profile for development, but it is not optimized with respect to properties which influence the persistence at the site of action or its selectivity over obvious off-target nuisances. As a design tool for optimization, a computational model was created based on the structure of Der p 1.0105 and on the structures of peptidic inhibitors bound to cathepsin K. Compound **7** was built and energy-minimized in the substrate-binding groove of Der p 1 with the electrophilic carbonyl of the pyruvamide moiety orientated to interact with C^34^ and the peptide backbone aligned to follow a trajectory similar to that of the other peptidic inhibitors. As shown in Fig. 5, this revealed interactions between the amide carbonyl of the pyruvamide and the backbone NH of C^114^, the NH of the P_1_ subunit, and the carbonyl of Y^249^ and the formation of a donor:acceptor pair with the backbone carbonyl and NH of D^154^. This tool enabled the prioritization of chemical design decisions which were rigorously explored by iterative rounds of synthesis and screening, details of which are provided elsewhere (Newton et al., 2014). Counterscreening against Der f 1 provided confidence that compounds designed using Der p 1 as template were similarly active in other HDM species.

**Fig. 6.**
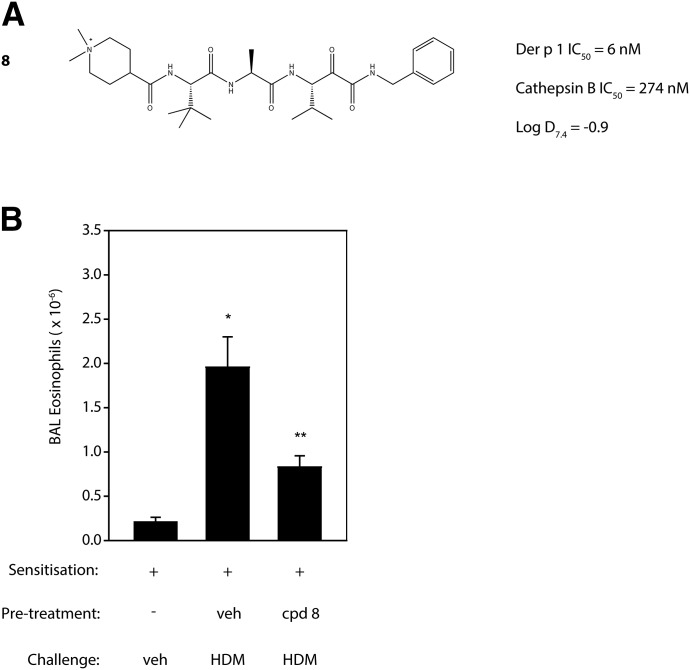
(A) Quaternary ammonium compound **8**. (B) Effect of a single aerosolized dose of **8** (18 nmol/kg) on the recruitment of eosinophils to the airways following aerosol challenge with mixed HDM allergens in sensitized brown Norway rats. The compound was administered 2 hours prior to HDM allergen by means of a Penn-Century aerosolizer (Penn-Century, Philadelphia, USA). Data are displayed as means ± S.E. in groups of 10 animals. *P < 0.05 vs. vehicle (veh) challenge; **P < 0.05 vs. HDM without test substance pretreatment; veh, vehicle. Full details of treatment protocols are provided by Newton et al. (2014) and the online supporting information, https://pubs.acs.org/doi/suppl/10.1021/jm501102h and https://pubs.acs.org/doi/suppl/10.1021/jm501102h/suppl_file/jm501102h_si_001.pdf. BAL, bronchoalveolar lavage.

Pursuant to producing compounds which were efficacious against real-life allergen exposure and not just a single purified allergen, a range of in vivo studies were undertaken to establish confidence in developability (Newton et al., 2014). An example for compound **8** is shown in Fig. 6. Eosinophils in bronchoalveolar lavage fluid were chosen as a readout in these studies because elevations in blood eosinophils and eosinophil recruitment to the airways are hallmark features of allergic asthma in many patients and correlate with disease severity. Importantly, these animals were sensitized and challenged with the complete palette of house dust mite allergens in a naturally derived mixture and not simply with the purified target allergen. As illustrated, a single inhaled dose of the compound produced a striking reduction in eosinophil recruitment after acute allergen challenge in an IgE-dependent model. This is an interesting result because our expectation is that ADIs will exhibit greatest benefits against underlying IgE-independent mechanisms which drive the acquisition and persistence of disease. Thus, even in a model where a strong IgE response existed and allergen challenge comprised a full palette of HDM allergens, targeting only the Der p 1 component produced a result suggesting useful efficacy (Newton et al., 2014).

**Fig. 7.**
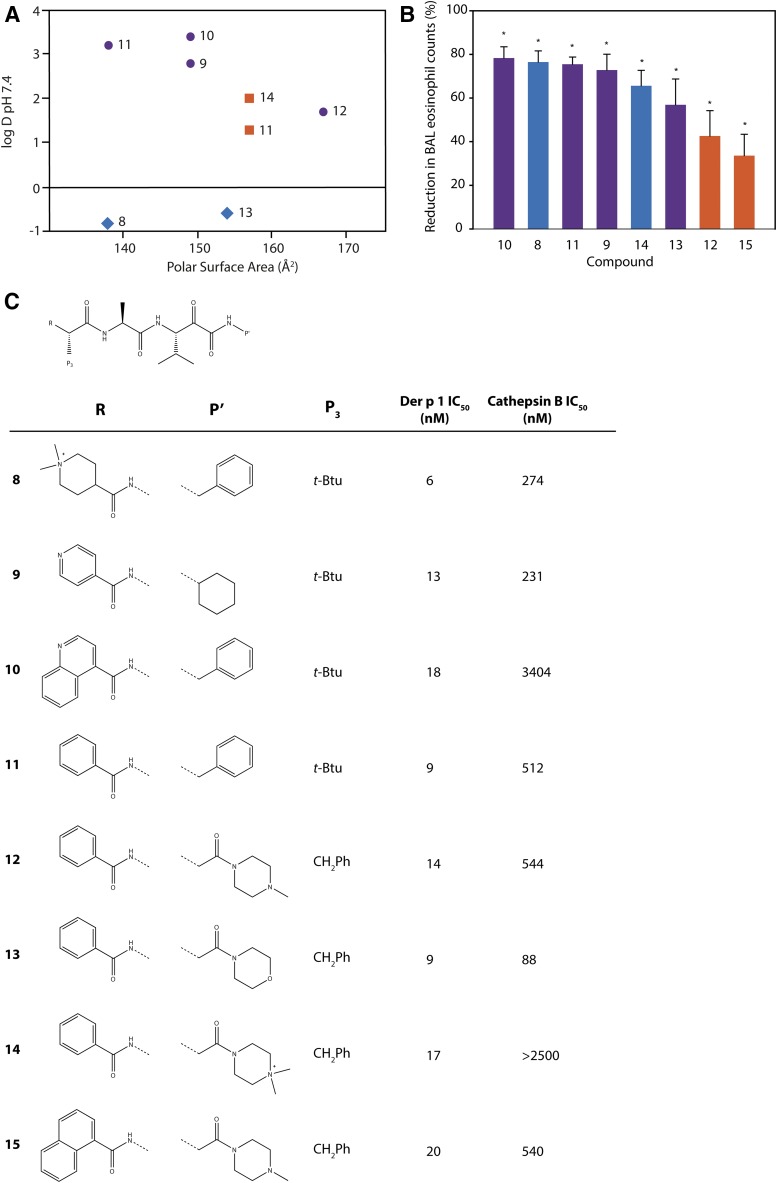
(A) Relationship between inhibitor polar surface area and log D_7.4_ for a subset of compounds used for the examination of in vivo efficacy. Symbols depict ionization state at pH 7.4: neutral (purple circles), positively charged basic center (orange squares), and positively charged quaternary ammonium (blue diamonds). (B) Percentage reduction in bronchoalveolar eosinophil counts 48 hours after challenge of nonsensitized rats with a natural mixture of HDM allergens following a single aerosolized dose of test substance administered 2 hours prior to allergen challenge. The percentage reduction was calculated relative to vehicle-pretreated animals which underwent similar HDM allergen challenge. Test substances were administered at a drug:target ratio of 50:1 with the exception of compound **11**, tested at 15:1. Data are the mean ± S.E. with 10 animals per treatment group. *P < 0.001 compared with HDM challenge control. Column coloration depicts ionization state of test substance as in (A). (C) Structural information and potency data for compounds used in this study. Full experimental details for chemical syntheses and biologic studies are provided by Newton et al. (2014) and the online supporting information, https://pubs.acs.org/doi/suppl/10.1021/jm501102h and https://pubs.acs.org/doi/suppl/10.1021/jm501102h/suppl_file/jm501102h_si_001.pdf. BAL, bronchoalveolar lavage.

To further explore the chemical features necessary for a persistence of effect, we evaluated a subset of potent compounds which were stable in contact with airway cells. To neutralize additional variables created in IgE-dependent challenge models, we electively conducted investigations using animals which were HDM allergen–naïve because aerosol challenge with Der p 1 or HDM allergen mixtures results in eosinophil recruitment to the airways in the absence of HDM sensitization (Newton et al., 2014). The kinetics of this IgE-independent innate response are indistinguishable from sensitized animals, but the effect is smaller in magnitude. Compounds selected for these studies spanned a range of log *D*_7.4_ values and polar surface areas (Fig. 7). Better efficacy was associated with lipophilicity, but good activity also existed in quaternary ammonium compounds (e.g., **8**, **14**) (Newton et al., 2014). As anticipated, the latter benefited from having low oral absorption, thus restricting systemic exposure in a manner which offers some advantages over alternate approaches where deliberately promoting the biotransformation of absorbed drugs can generate unexpected metabolic liabilities.

**Fig. 8.**
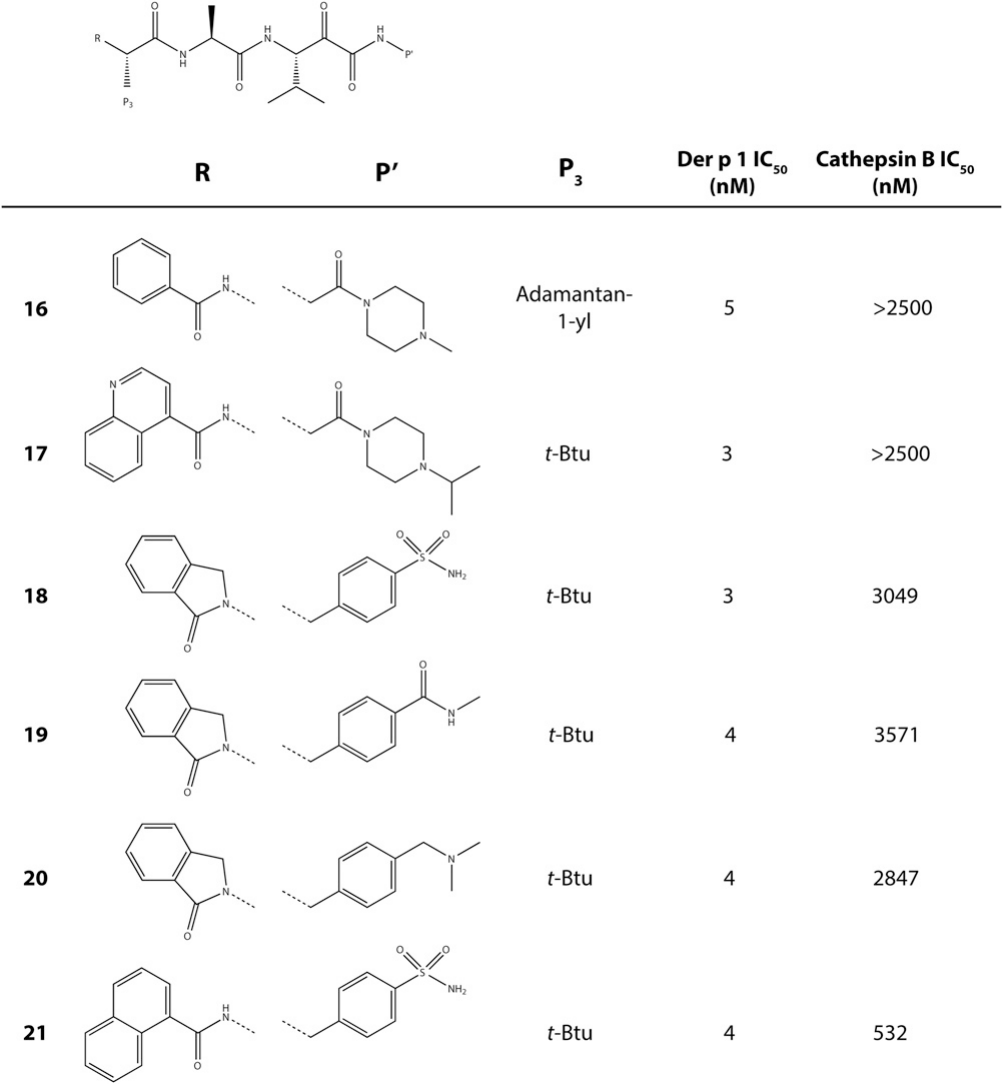
Further optimization of the P_4_ (R) and P′ groups produces compounds showing high target potency and intrinsic selectivity over cathepsin B. Chemical syntheses and enzymatic activity assays are described by Newton et al. (2014) and the online supporting information, https://pubs.acs.org/doi/suppl/10.1021/jm501102h and https://pubs.acs.org/doi/suppl/10.1021/jm501102h/suppl_file/jm501102h_si_001.pdf.

As the electrophilic pyruvamide motif has potential for nonspecific interaction with suitable nucleophiles, it was imperative to achieve selectivity over potential off-targets, even though many of these are intracellular and thus intrinsically resilient to reaction compared with the facile extracellular interaction with Der p 1. As already described, the S_3_ pocket of Der p 1 is more capacious than in cathepsins, and our screening campaign confirmed that the steric bulk in this position increased selectivity while retaining potency on target. Inspection of the structural model suggested that the S_2_ pocket of Der p 1 was shallow compared with cathepsin B, K, or S, and so with compact substituents at this position, it was possible to obtain a useful balance of potency and selectivity (Newton et al., 2014). As an aside, Blo t 1 exhibits a similar feature at its putative S_2_ pocket, suggesting that compounds designed against a template from a pervasive HDM species may have characteristics which translate to allergens from mites whose evolution has diverged to suit niche environments. Once confidence in the contributions of the P_2_ and P_3_ substituents were obtained, we explored greater variation in the P_4_ and P′ groups, which pleasingly revealed the feasibility of synthesizing compounds, such as **16–21**, of high inhibitory potency and good selectivity consistent with developability (Fig. 8) (Newton et al., 2014).

**Fig. 9.**
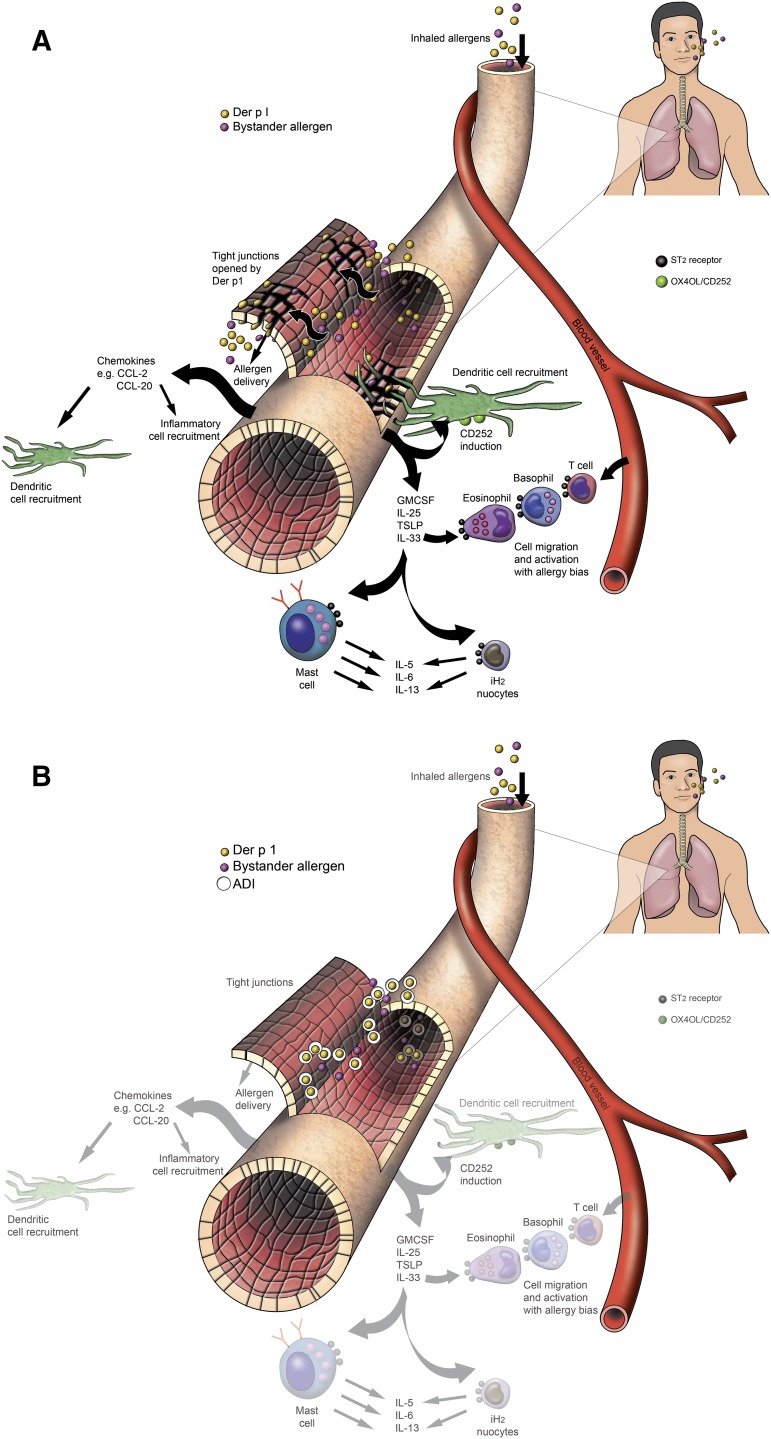
Predicted effects profiles for ADIs based on preclinical discovery research models (published and on-file data). (A) Effects of HDM exposure in the airways with a focus on innate immune responses. (B) Example effects of HDM exposure in the presence of ADI drug, with affected pathways grayed out. GMCSF, granulocyte macrophage-colony stimulating factor.

We examined exemplars of optimized compounds in vivo using a variety of readouts to establish confidence in efficacy and duration of protection. These studies confirmed the structural features required for compliance with the exacting candidate drug template and provided test data from which clinical candidates could be selected (Newton et al., 2014). A wider examination of the effects of ADIs in experimental models reveals the potential for a fascinating spectrum of effects in the clinic and a profile which is interestingly differentiated from the current standard of care and other entities in development for asthma (Fig. 9; Table 3). The overall impression is of an intervention which has the breadth normally associated with corticosteroids. However, ADIs can affect events which are not directly regulated by steroids, and notably, their broad range of effects is achieved without incurring penalties from nonspecific immunosuppression. Pre-eminent in the readouts which show positive effects of ADIs are those strongly associated with events identified by experimental biology as being indispensable in allergy. Some of the signaling entities which drive these indispensable events are now the target of biologic therapies in development. The ability to attenuate the same signals simultaneously by means of an inhaled small molecule provides a superior option with the potential for usage across a broader spectrum of patients.

**TABLE 3:** Potential strategic positioning of ADIs in allergic asthma versus alternative approaches

Intervention	Examples	Remarks
Allergen delivery inhibitors	Group 1 HDM protease allergen inhibitors	Small molecule
		Inhaled delivery to target organ
		Attractive profile (nonhuman target, extracellular action) with disease modification
		Root cause–directed
		Potential to prevent exacerbations
		Mechanistic differentiation
		Potential addition to standard of care at all levels of disease severity
		Potentially prescribable at nonspecialist level
		Low cost of goods compared with biologics
		Exploitable as combination therapy and/or other conditions
Alternative small molecules in discovery/development	Downstream signal transduction and effector mechanisms—various targets	Uncertain potential to surpass or add significantly to inhaled steroids
		Multiple redundant effector pathways are confounders of efficacy
		Potentially prescribable at nonspecialist level
		Low cost of goods compared with biologics
Biologics—approved or in development	Anticytokine mAbs	High cost of goods
	Antireceptor mAbs	Mainly applicable to severe disease only
	Anti-IgE mAbs	Inconvenient to use
	Anti-IgE vaccine (pAb)	Multiple redundant pathways are confounders of efficacy
		High safety barriers (esp. IgE vaccine)
		Specialist use only
		Patchy targeting of innate pathways
Immunotherapy	Allergen-specific immunotherapy Immune deviation	Moderately high cost of goods
		Can be inconvenient to use
		Specialists must be involved in GP use
		Chronic safety of immune deviation is unproven
		Poor targeting of key innate pathways

GP, general practitioner; mAb, monoclonal antibody; pAb, polyclonal antibody.

## Concluding Remarks

From the earliest scientific investigations of allergy, the causative link between allergens and the diseases they produce has attracted attention as a means of disease treatment. The outstanding example of this is the pioneering work of Freeman and Noon (1911), which led to the development of allergen immunotherapy and attempted to tolerize the immune system to a particular allergen. However, targets for small-molecule interventions at the root cause level have been nonobvious until recently, so the pharmacotherapy of allergy adopted a different approach which exploited progress in the identification of bioactive mediators and the elucidation of effector mechanisms. The renaissance of innate immunity and an increasing understanding of the molecular basis of allergenicity now creates the first opportunity for pharmacotherapy to target the causative agents of many allergic diseases and thereby prevent the activation of sentinel mechanisms which initiate and sustain disease. In this review, we described progress toward that ambitious objective for what is arguably the most significant of all indoor allergens. The power of the intervention exploits the growing awareness that the allergome is not a molecular democracy: an exclusive cadre of initiator allergens play decisive roles in driving disease. This vulnerability creates a pharmacologic opportunity to exert a broad spectrum of benefits by careful selection of target and chemical design, as exemplified by the pioneer work with ADIs. Naturally, it will be of considerable interest to follow the progression into the clinic of the new drugs from this unique program.

## Abbreviations

ADAM: A disintegrin and metalloprotease
ADI: allergen delivery inhibitor
ADZ 50,000: (S)-3-((S)-2-((S)-2-benzamido-3-methylbutanamido)propanamido)-2-oxoheptyl-2,6-bis(trifluoromethyl)benzoate
Blo t: *Blomia tropicalis*
CCL: C-C chemokine ligand
CD: cluster of differentiation
DC: dendritic cell
DC-SIGN: dendritic cell–specific intercellular adhesion molecule 3–grabbing nonintegrin
Der f: *Dermatopagoides farinae*
Der p: *Dermatophagoides pteronyssinus*
DPI: dry powder inhaler
EMT: epithelial-mesenchymal transition
HDM: house dust mite
IL: interleukin
LPS: lipopolysaccharide
MD-2: myeloid differentiation protein-2
MRGPR: Mas-related G-protein coupled receptor
n: native form
Nrf2: nuclear factor (erythroid-derived 2)-like 2
PAR: protease-activated receptor
r: recombinant protein engineering
ROS: reactive oxygen species
siRNA: small interfering RNA
TAK-242: ethyl-(6*R*)-6-(*N*-(2-chloro-4-fluorophenyl)sulfamoyl)cyclohex-1-ene-1-carboxylate
Th2: type 2 T-helper cell
TJ: tight junction
TLR: Toll-like receptor
TSLP: thymic stromal lymphopoietin
